# Respiratory and other organ manifestations in *NKX2-1*-related disorders: a systematic review

**DOI:** 10.3389/fmed.2025.1507513

**Published:** 2025-05-06

**Authors:** Katarzyna Michel, Alba Ruiz-Ramos, Laia Nou-Fontanet, Carmen Martín-Gomez, Beatriz Carmona Hidalgo, Rebeca Isabel-Gomez, María Piedad Rosario-Lozano, Rocio Rodriguez-Lopez, Thomas O. F. Wagner, Juan Antonio Blasco-Amaro, Matthias Griese, Juan Darío Ortigoza-Escobar

**Affiliations:** ^1^Hauner Children's Hospital, Ludwig Maximilians University, German Center for Lung Research (DZL), Munich, Germany; ^2^European Reference Network for Rare Diseases of the Respiratory System (ERN-LUNG), Munich, Germany; ^3^Health Technology Assessment Area-AETSA, Andalusian Public Foundation for Progress and Health (“Fundación Progreso y Salud”–“FPS”), Seville, Spain; ^4^Movement Disorders Unit, Department of Child Neurology, Institut de Recerca Sant Joan de Déu, Barcelona, Spain; ^5^Research Group HUM604: Lifestyle Development in the Life Cycle and Health Promotion, University of Huelva, Huelva, Spain; ^6^Frankfurter Referenzzentrum für Seltene Erkrankungen (FRZSE), Universitätsklinikum Frankfurt am Main, Wolfgang von Goethe-Universität, Munich, Germany; ^7^U-703 Centre for Biomedical Research on Rare Diseases (CIBER-ER), Instituto de Salud Carlos III, Barcelona, Spain; ^8^European Reference Network for Rare Neurological Diseases (ERN-RND), Tübingen, Germany

**Keywords:** *NKX2-1*, benign hereditary chorea, interstitial lung disease, neonatal respiratory distress, asthma, recurrent respiratory infection, systematic review, brain-thyroid-lung syndrome

## Abstract

**Background:**

*NKX2-1*-related disorders (*NKX2-1*-RD) encompass a spectrum of conditions arising from pathogenic deletions or variants in the *NKX2-1* gene, crucial for thyroid, lung, and brain development. Respiratory manifestations in *NKX2-1*-RD range from neonatal respiratory distress to severe lung diseases, constituting a leading cause of mortality. This study will review and synthesize NKX2-1-RD respiratory phenotypes, genetic alterations, and long-term trajectories.

**Methods:**

We conducted a systematic review using PRISMA and PICO question formats. From January 2002 to July 2023, major biomedical databases and rare disease resources were searched. Genetically confirmed *NKX2-1*-RD patients with respiratory symptoms were eligible for this study.

**Results:**

Out of 4,569 studies, 38 met inclusion criteria, predominantly case reports and series. The study included 148 patients, revealing diverse respiratory phenotypes and treatments. Respiratory manifestations emerged at various ages, with neonatal respiratory distress, asthma, interstitial lung disease, and lung cancer observed. Nonsense mutations in *NKX2-1* were linked to lung cancer. Treatment varied, including oxygen supplementation, ventilation, antibiotics, and lung transplantation. Long-term follow-up disclosed heterogeneous respiratory outcomes, with some patients asymptomatic while others faced chronic insufficiency or recurrent infections. The overall survival of informed cases was about 60%.

**Conclusion:**

This study highlights the complex respiratory manifestations of *NKX2-1*-RD and their impact on patient outcomes. The findings support standardized respiratory follow-up protocols and provide clinical management insights despite study quality and sample size limitations. We discuss the challenges of treating diverse respiratory conditions in this rare clinical entity and lay the groundwork for future research.

## Introduction

*NKX2-1*-related disorders (*NKX2-1*-RD, OMIM#610978) refer to a group of disorders associated with pathogenic deletions or variants in the *NKX2-1* gene. Formerly identified as thyroid transcription factor 1 (TTF-1), the *NKX2-1* gene plays a pivotal role in organ development, impacting the thyroid, lung, and brain. Specifically, *NKX2-1* orchestrates pulmonary development by influencing lung patterns, including proximal airway structures (1), and modulating genes involved in epithelial cell fate determination (2–4). This regulatory function extends to the activation of lung-specific genes such as SP-A (5), SP-B (6), and SP-C (7), encoding crucial pulmonary surfactant proteins. Additionally, *NKX2-1* involvement in lung cancer pathogenesis (8) underscores its significance as a biomarker for distinguishing primary lung adenocarcinomas from other pulmonary conditions (9, 10).

Respiratory manifestations linked to *NKX2-1*-RD encompass a broad spectrum, including interstitial lung disease (ILD), neonatal respiratory distress syndrome (RDS), recurrent infections, chronic respiratory insufficiency, pulmonary hypertension, asthma, and lung cancer. Understanding this range of respiratory manifestations is pivotal for recognizing diverse clinical presentations, facilitating accurate diagnoses, and customizing effective management strategies. Furthermore, this comprehension significantly contributes to ongoing scientific endeavors and the development of therapeutic interventions. Of particular note is the observation that respiratory mortality in *NKX2-1*-RD is primarily attributed to neonatal respiratory distress syndrome, interstitial lung disease, and pulmonary fibrosis (11).

Recognizing the gravity of these respiratory implications, the European Reference Network for Rare Neurological Disorders (ERN-RND), in collaboration with ERN-Lung, is actively engaged in establishing inaugural clinical practice guidelines for *NKX2-1*-RD. This initiative, part of the broader European Program ERN Guidelines, aspires to provide an up-to-date synthesis of available evidence on respiratory manifestations associated with *NKX2-1*-RD.

## Methods

This study comprises systematic reviews of two research questions reported following the Preferred Reporting Items for Systematic Review and Meta-Analyses (PRISMA) statement (12).

### Research question

The PICO (Population-Intervention-Comparator-Outcome) question format was employed to guide the literature search, posing the following inquiries: *What are the best procedures for the diagnosis of lung diseases in patients with NKX2-1-related disorders?* and *What are the best procedures for treatment and follow-up of lung diseases in patients with NKX2-1-related disorders?* (Supplementary Data 1). These questions covered all the aspects related to the detection, diagnosis, treatment, and follow-up of respiratory manifestations in *NKX2-1*-RD. The protocols of the systematic reviews for each question were previously registered in the International Prospective Register of Systematic Reviews (PROSPERO) repository with the identifications CRD42023448437 and CRD42023448440.

### Eligibility criteria

Specific inclusion criteria were employed to select the relevant studies for these systematic reviews. The population criteria were common for both research questions: patients of all ages with genetic confirmation of the disease (variants in *NKX2-1*, previously known as *TTF-1,* or deletion in the 14q13.3 chromosome) with respiratory manifestations. Non-human studies and patients without genetic confirmation of the disease were excluded. Given the rare condition, any comparator was used.

The following specifications apply to the interventions and their outcomes: For diagnosis, the interventions included physical examination, pulse oximetry test, blood tests (blood gases, hemogram, reactive C protein, blood culture, etc.), pulmonary function testing (spirometry, bodyplethysmography), exercise tolerance testing, diffusion capacity for carbon monoxide (DLCO), imaging (chest X-ray, CT scan, PET-CT scan), lung biopsy (transbronchial forceps- or cryobiopsy, open- or thoracoscopic biopsy), and outcomes were determined based on test results related to the diagnosis. Regarding treatment, the interventions comprised supplementary oxygen, noninvasive and invasive respiratory support, medication (bronchodilators, corticosteroids, immunosuppressive therapy, antibiotics, exogenous surfactant, etc.), chest physiotherapy, lung transplant, cancer surgery, radiotherapy, chemotherapy, and supportive care, including nutrition, immunization, and environmental care. Outcomes were assessed in terms of the indication for hospitalization, treatment effectiveness, adverse effects of treatments, treatment in special situations (pregnancy), and follow-up (lung outcomes, patient and professional education, quality of life, and self-management in adulthood).

The included study designs were primary studies, systematic reviews, and randomized controlled trials (RCTs). Narrative reviews, protocols, conference articles, editorials, letters to the editor, and those whose full text was unavailable to be retrieved were excluded. While the inclusion criteria allowed articles in all languages, all retrieved articles were written in English.

### Search strategy

A systematic literature search was carried out for each research question to review the scientific evidence. MEDLINE (Ovid), Embase (Elsevier), the Cochrane Library (Wiley), APA PsycInfo (EBSCO), CINAHL Complete (EBSCO), the Web of Science (WOS), the TRIP PRO medical database, and the International Health Technology Assessment (INAHTA) database were searched as reference databases, and additional databases were included to retrieve gray literature such as the Cochrane Controlled Register of Trials (CENTRAL) and subject-specific information resources on rare diseases, Orphanet, EURORDIS, NORD, RARE-Best Practices, and Gene Reviews.

The searches covered the period from January 2002 to August 2022, and were updated in July 2023. The included records date back to 2002, when the *NKX2-1* gene variants were first discovered and named. Both controlled language (descriptors) and free terminology were used to search for studies. The initial strategies were carried out in MEDLINE (Ovid) and later adapted to each database’s syntax. The Medline searches for each systematic review are available in Supplementary Data 8.

### Study screening and data extraction

The identified references were imported into the reference management section of the Covidence Systematic Review Software (Veritas Health Innovation; 2023, https://www.covidence.org/), and the duplicates were automatically removed. A different review was created for each question. Two authors independently filtered the references by title, abstract, and full text (KM, ARR) according to the selection criteria. The disagreements between the reviewers were resolved by a third researcher (JDOE). The data were extracted by two independent authors (KM, ARR) and recorded in Excel spreadsheets. Detailed information from the studies, including authorship, publication year, study design, and location, was systematically extracted. Furthermore, specific clinical data for each patient, encompassing age, gender, results of genetic tests, pulmonary diagnosis, diagnostic procedures, phenotype, treatment modalities, follow-up information, and other relevant data, were meticulously documented.

### Quality assessment

The methodological quality assessment of each study was conducted independently by two authors (KM, ARR), and discrepancies in individual assessments were resolved through discussion to achieve consensus. Specific tools tailored to each study type were employed for quality assessment. The identified references were categorized as case reports, case series, and cohort studies. Case reports and case series were assessed using the tool developed by Murad et al. (13) that evaluates methodological quality in low-evidence studies crucial for decision-making. The tool consists of eight items across four domains (selection, ascertainment, causality, and reporting). Article quality was rated based on the sum of binary responses (1 = low risk of bias, 0 = high risk of bias) for each item, resulting in a score ranging from 0 (poor quality) to 8 (high quality) (14). Adaptations to the ascertainment, causality, and reporting items were made to suit the specific clinical scenario addressed in the research questions.

## Results

### Study selection

During the initial search, a total of 4,576 records were located. After removing duplicates, 4,560 potentially relevant studies remained. Title and abstract screening were carried out, yielding 131 studies. Nine references were added after specialist consultants (15–24). While conducting additional *NKX2-1* reviews addressing different questions than those posed by the respiratory PICO, the authors observed that the systematic search did not retrieve these nine studies. These studies were included later due to their focus on respiratory symptoms of the disease, which, although important, were not the primary target of the initial search strategy. These studies primarily addressed other affected systems, and it is likely that they were not indexed under the search terms we used, which is why they were not retrieved in the initial search. After further evaluation, it was determined that these studies included patients with respiratory symptoms, justifying their inclusion.

**Figure 1:**
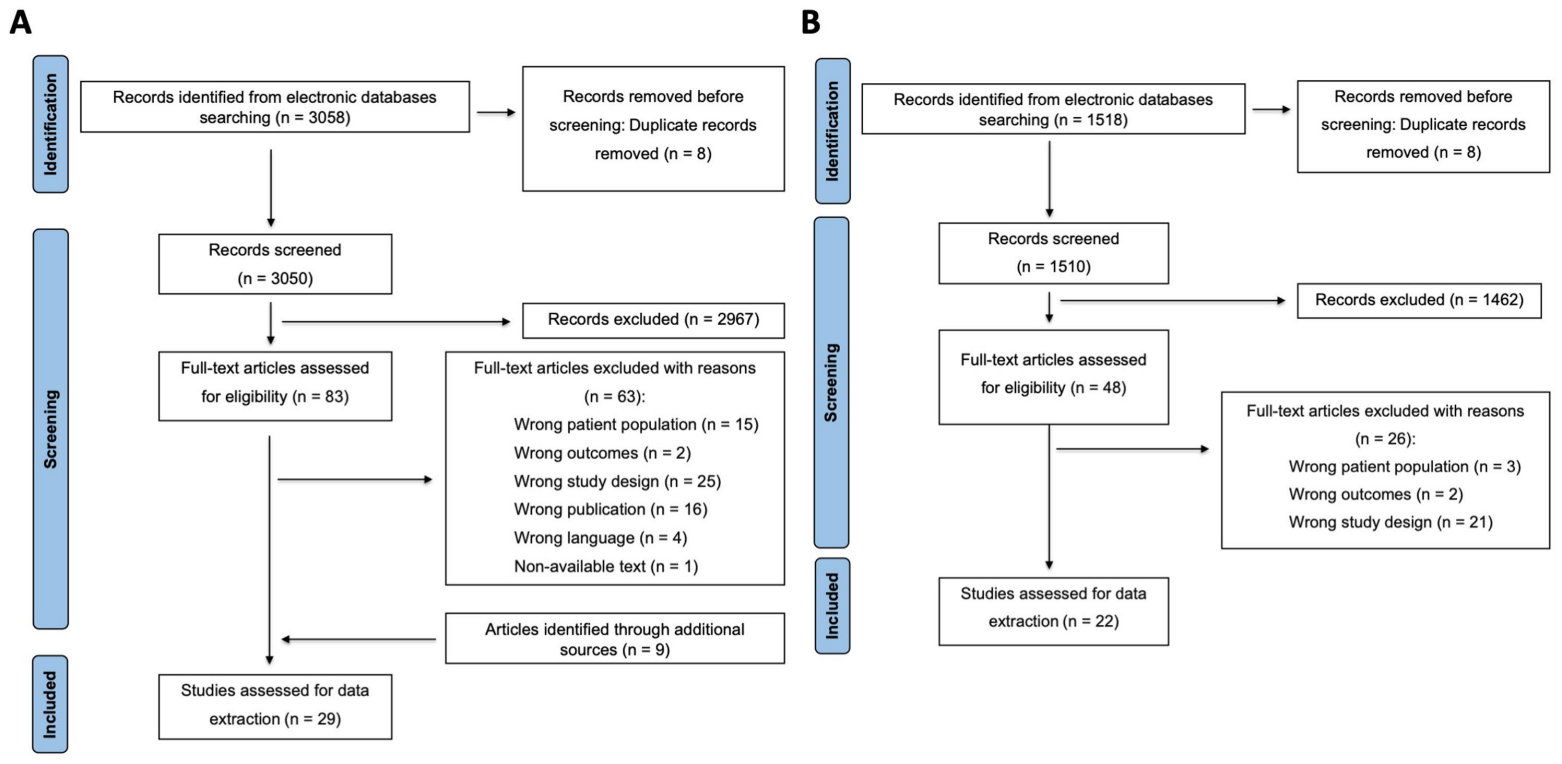
Illustrates the PRISMA flowchart depicting the title-abstract and full-text screening of the articles related to the respiratory manifestations in patients with *NKX2-1-*RD. **(A)** *What are the best procedures for the diagnosis of lung diseases in patients with NKX2-1-related disorders?* **(B)** *What are the best procedures for treatment and follow-up of lung diseases in patients with NKX2-1-related disorders?*

According to inclusion and exclusion criteria, 51 studies were included following full-text screening. 13 included studies were shared by the two PICO questions. Therefore, 38 studies were finally analyzed (15–23, 25–53). Additionally, three references (24, 54, 55) were initially included in the PICO questions. However, during data extraction, the reviewers identified that these studies referred to patients already included in other studies. The two independent screening processes (PRISMA flowcharts) are depicted in Figures 1A,B. The list of included and excluded articles after full-text review, along with the reasons for their exclusion, is shown in Supplementary Data 2. The excluded studies were classified according to the first criterion that did not fit the PICO format.

### Study characteristics

44.7% of the included studies were case series (*n* = 17), and 55.3% were case reports (*n* = 21). No cohort studies were identified. Due to the rare clinical condition studied, no anticipated randomized controlled trials or systematic reviews were available. 44.7% of the data originated from Europe (*n* = 17), 44.7% from America (*n* = 17), 7.9% from Asia (*n* = 3), and 2.6% from Australia (*n* = 1) (Supplementary Data 3).

### Patients’ demographics and respiratory manifestations

**Figure 2:**
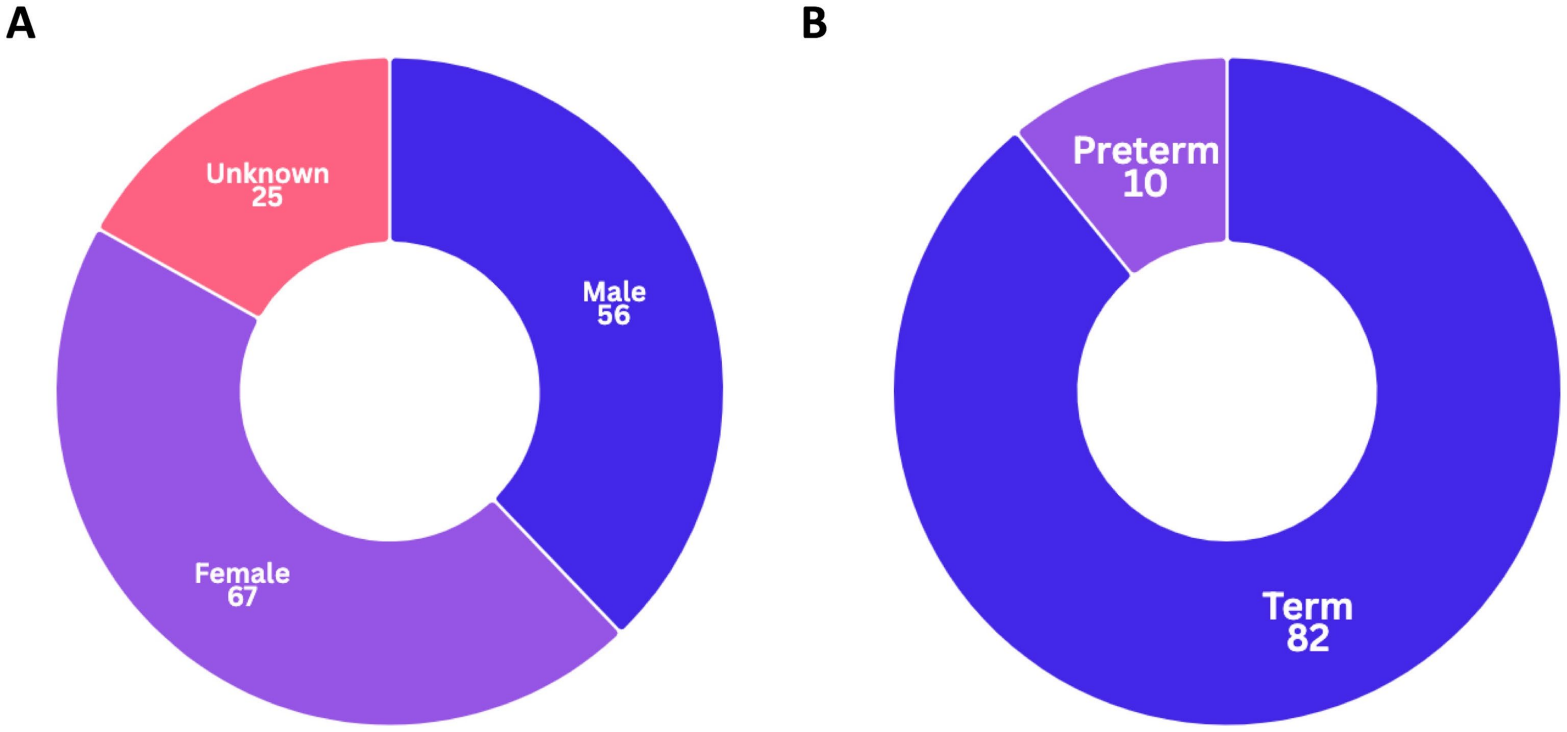
Distribution of **(A)** sex and **(B)** gestational age classification among the studied patients.

The data were collected from 148 patients, comprising 56 males and 67 females, and 25 individuals with unknown sex (Figure 2A; Supplementary Data 4). Among the 92 cases with available gestational age data, 82 were classified as term and 10 as preterm (Figure 2B). Newborn screening for hypothyroidism was available in 115 out of 148 patients, with 20 testing positive and 95 negative. Respiratory symptoms typically manifest at a mean age of 4.4 years, with a median of 30 days (neonatal period), spanning from birth to 73 years.

The initial respiratory symptoms were diverse, including neonatal respiratory distress syndrome (RDS) in 70 patients, asthma and/or wheezing in 20 patients, ILD (with or without hypoxemia), including pulmonary fibrosis in nine patients, severe or recurrent infection with chronic cough in 7 patients, tachypnea and/or hypoxemia in 13 patients, and lung cancer in 1 patient.

In relation to the NKX2-1-RD phenotype, diverse clinical presentations were observed within the studied cohort, both at diagnosis and throughout the disease course. All 148 patients had pulmonary involvement; this was exclusive in only 15 patients, while five showed pulmonary and endocrinological manifestations. Additionally, 19 patients displayed a combination of pulmonary and neurological involvement. The most prevalent category encompassed patients with pulmonary, endocrinological, and neurological manifestations, totaling 86 individuals with this set of phenotypes. 23 subjects had incomplete information that prevented a precise determination of their phenotype, and 4 additional patients were excluded due to issues with the available data. These patients were not included in the detailed analysis. Similarly, in the following, we use only the informative cases available as the denominator.

### Phenotypic respiratory diversity and other organ involvement in *NKX2-1*-RD

**Figure 3:**
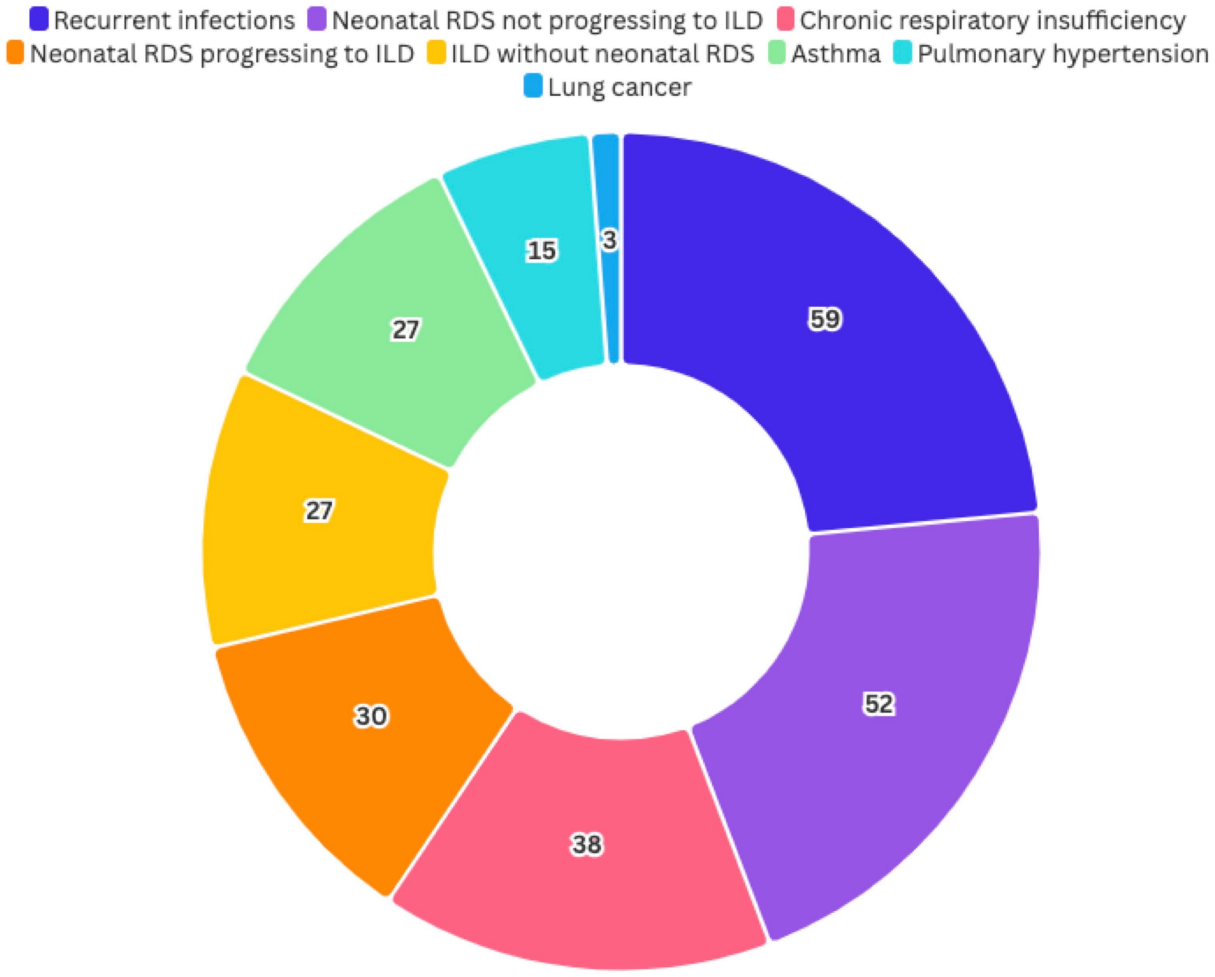
Distribution of patients across various respiratory phenotypes, highlighting the presence of conditions such as neonatal respiratory distress syndrome (RDS), interstitial lung disease (ILD), recurrent infections, chronic respiratory insufficiency, pulmonary hypertension, asthma, and lung cancer.

**Figure 4:**
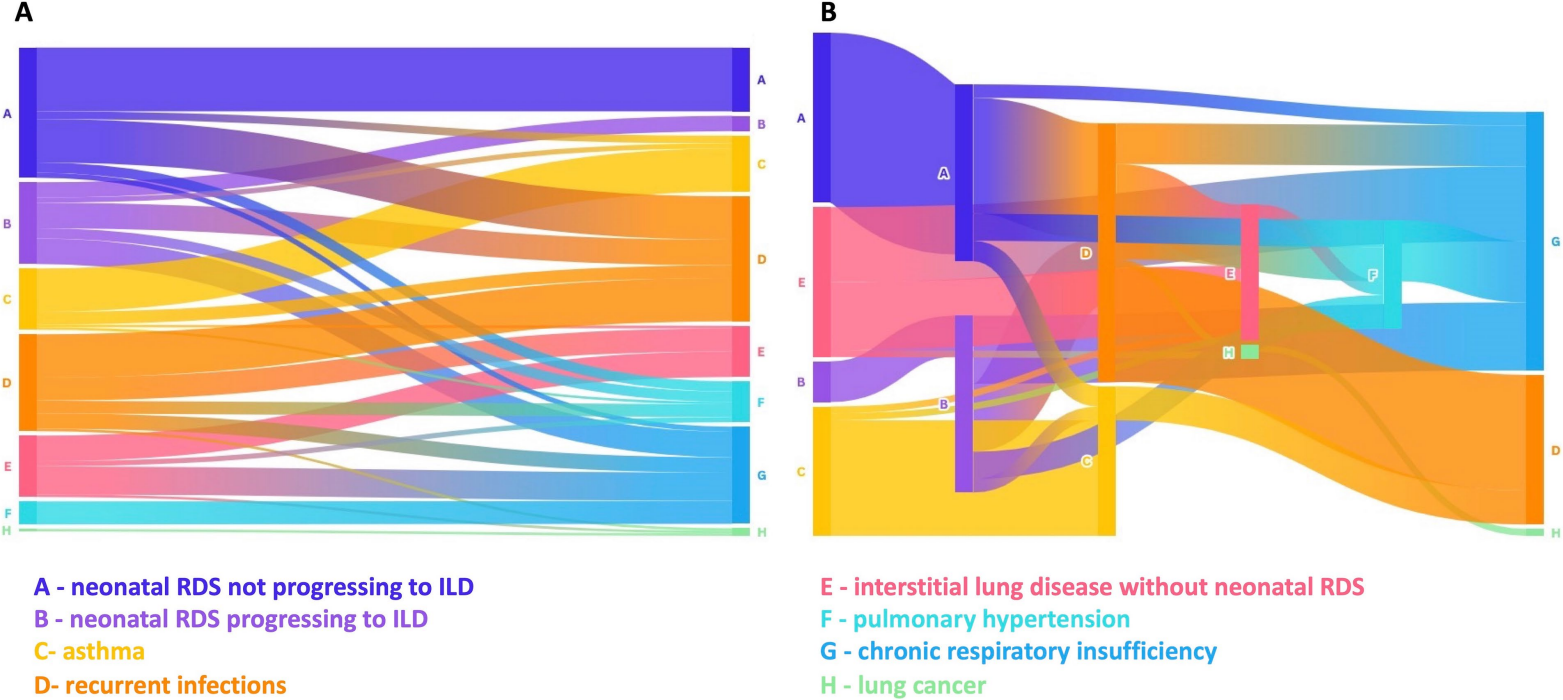
**(A)** An alluvial diagram illustrating the transition of patients from one respiratory phenotype to another over time. **(B)** Multi-step alluvial diagram depicting cases with complex trajectories, where patients exhibited multiple respiratory phenotypes throughout their clinical course.

*NKX2-1*-RD demonstrates phenotypic respiratory diversity along with endocrinological and neurological involvement. Among the patients, neonatal RDS not progressing to ILD was observed in 52 out of 143 cases (male/female 17:25, unknown 10), while neonatal RDS progressing to ILD was noted in 30 out of 140 patients (male/female 19:9, unknown 2). The difference in sex distribution between these groups was statistically significant (*χ*^2^ = 4.01, *p* = 0.045; Fisher’s exact test *p* = 0.030), suggesting a potential association between male sex and the progression to ILD. Recurrent infections were reported in 59 out of 99 cases (male/female 15:23, unknown 21), and ILD without neonatal RDS was present in 27 out of 142 patients (male/female 8:19). Chronic respiratory insufficiency occurred in 38 out of 123 cases (male/female 19:19), pulmonary hypertension in 15 out of 135 patients (male/female 7:8), and asthma, recurrent bronchial obstruction, or wheeze in 27 out of 133 cases (male/female 12:11, unknown 4). Additionally, lung cancer was observed in three out of 115 patients (male/female, 1:2) (Figure 3). The alluvial diagram and the multi-step alluvial diagram in Figures 4A, B illustrate the interplay of distinct phenotypes in patients exhibiting respiratory manifestations and variants or deletions in *NKX2-1*.

Endocrinological involvement was present in 90 out of 131 cases, and neurological involvement was reported in a substantial 104 out of 124 cases.

Among the 15 cases with possibly exclusive respiratory manifestations, the distribution of respiratory phenotypes is presented in Table 1.

**Table 1:** Distribution of respiratory phenotypes in patients with exclusively respiratory symptoms (without thyroid or neurological involvement).

Patient	Neonatal RDS not progressing to ILD	Neonatal RDS progressing to ILD	Recurrent infections	Interstitial lung disease without neonatal RDS	Chronic respiratory insufficiency	Pulmonary hypertension	Asthma	Lung cancer
11	−	−	NA	+	+	−	−	NA
12	−	−	NA	+	+	−	−	NA
15	−	−	NA	+	NA	−	−	NA
16	−	+	NA	−	+	−	−	NA
51	−	−	−	+	+	−	−	−
52	−	−	−	+	+	+	−	−
53	−	−	−	+	NA	NA	NA	NA
54	−	+	+	−	NA	−	−	−
55	−	+	+	−	+	−	−	−
70	−	−	−	+	+	−	−	−
107	+	−	+	−	−	−	−	−
110	+	−	−	−	−	−	−	−
122	−	−	−	−	−	−	+	−
123	−	−	+	−	−	−	−	−
124	−	−	−	−	−	−	−	−

The table presents the presence or absence of various respiratory conditions in each patient, including neonatal respiratory distress syndrome (RDS), interstitial lung disease (ILD), recurrent infections, chronic respiratory insufficiency, pulmonary hypertension, asthma, and lung cancer. Patients with missing data are indicated as NA.

### Respiratory features observed across imaging, BAL, and biopsy assessments

For a very small fraction of patients, detailed respiratory information (including imaging) was available. Chest radiography (CR) was available for 10 out of 148 patients, with six showing diffuse bilateral opacities and three displaying bilateral opacities alongside other abnormalities such as infiltrates, pneumothorax, or atelectasis, and one case presented with parahilar infiltrates.

CT scans were reported for 34 out of 148 patients; focal consolidations were identified in 12 cases; and ground glass opacities were evident in 24 cases. Additionally, septal or pleural thickening, cystic lesions, bronchiectasis, and architectural distortion were each observed in four cases. Features consistent with usual interstitial pneumonia and a fibrosing interstitial pattern were present in three cases each. Furthermore, two cases exhibited nodular opacities, hilar adenopathy, and paraseptal emphysema.

Regarding pulmonary function test results, nine patients had detailed outcomes (Supplementary Data 5). All patients were older than 6 years (mean 32.2 years, median 25 years, range 8–61 years). Among the patients with results, the pulmonary phenotype included interstitial lung disease without neonatal RDS in 8 patients, recurrent infections in 4 patients, chronic respiratory insufficiency in 3 patients, neonatal RDS progressing to ILD in 1 patient, and lung cancer in 1 patient.

In addition to the pulmonary function tests, bronchoalveolar lavage (BAL) analyses were reported in 11 out of 148 patients; findings revealed neutrophilic inflammation in five cases, decreased levels of surfactant proteins B (SP-B) and C (SP-C) in two cases, and lymphocytic inflammation in one case.

16 histopathological descriptions of lung biopsies were conducted, two of which were in the context of autopsies (at 28 and 40 days of life). The average age, median, and age range of the histopathological study were 14.15 years, 0.75 years, and a range of 23 days to 64 years, respectively. The lung biopsies exhibited a spectrum of histopathological findings, including alveolar growth abnormalities in 9 cases, increased alveolar macrophages in 8 cases, type II cell hyperplasia in 8 cases, alveolar filling with PAS-positive material in 5 cases, alveolar wall thickening and remodeling in 4 cases, pulmonary hypertensive arteriopathy, interstitial pneumonia in 3 cases, foamy macrophages in 2 cases, emphysema, interstitial fibrosis, chronic inflammation, interstitial lymphocytic infiltrate, neuroendocrine cell hyperplasia (via bombesin stain), and non-specific interstitial pneumonia in 1 case each.

### Genetic analysis of alterations in the *NKX2-1* gene

**Figure 5:**
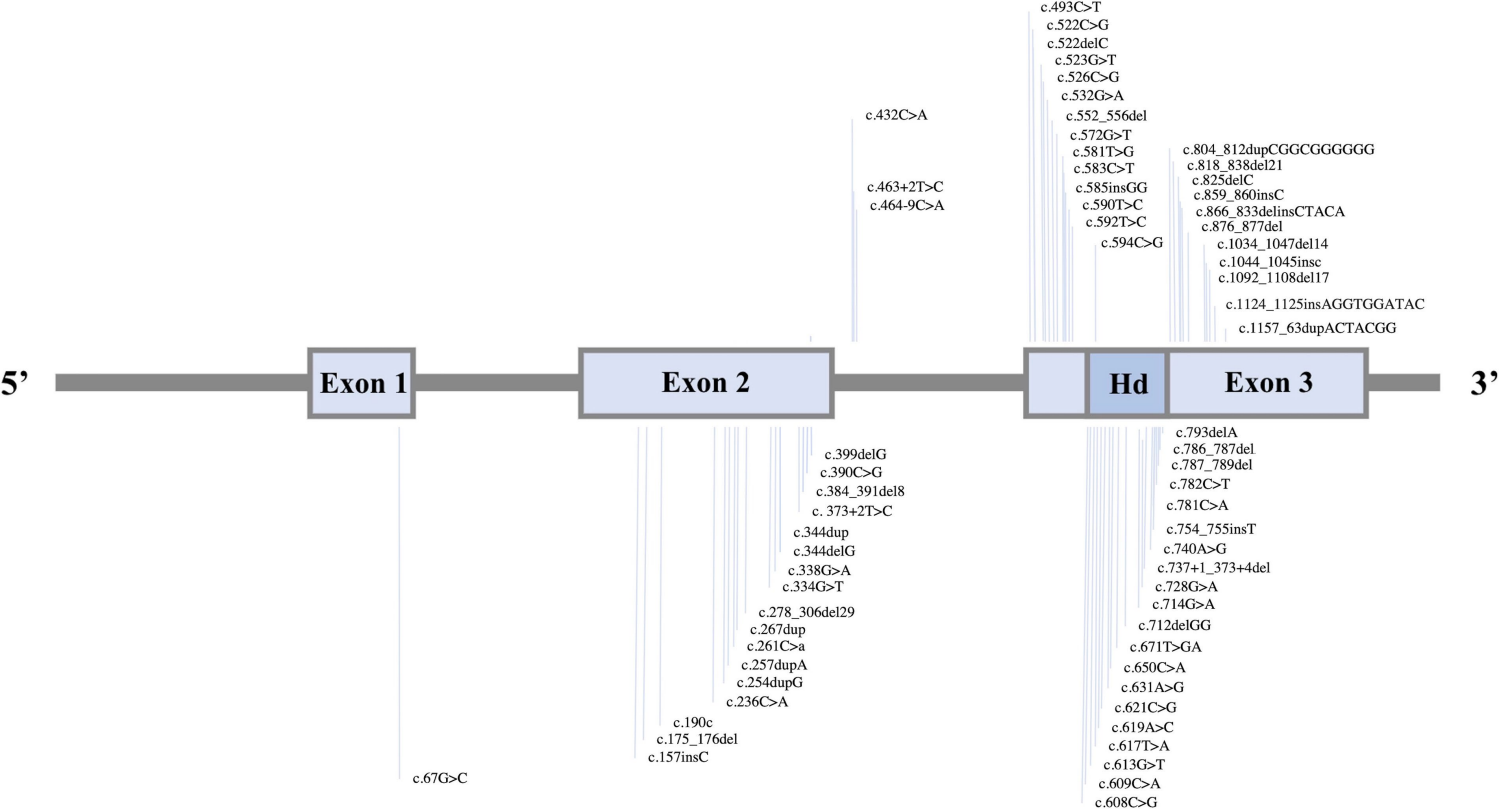
Distribution of 67 *NKX2-1* variants with respect to the functional domains of *NKX2-1* isoform 2 (RefSeq NM_003317.3). Distal and proximal promoters are indicated by arrows. Functional domains are shown in dark grey: tn (TIN domain), hd (homeodomain), and nk2 (NK2 domain).

**Figure 6:**
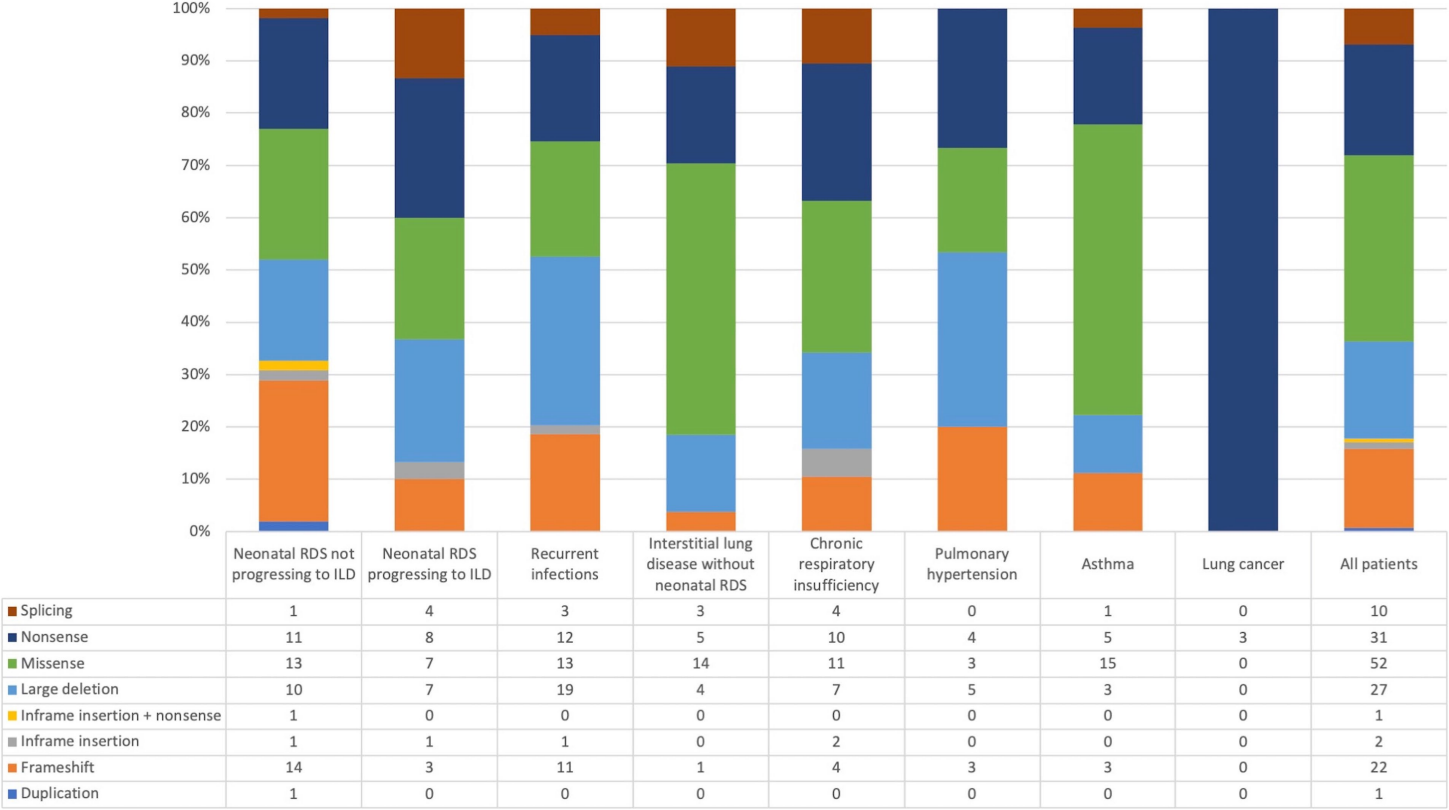
Distribution of patients across respiratory phenotypes and types of variants or deletions in the *NKX2-1* gene.

In the genetic analysis of patients, a total of 120 variants and 27 deletions in the *NKX2-1* gene were reported (Figure 5). The types of variants, listed in order of frequency, included missense (52 cases), nonsense (31 cases), frameshift (17 cases), splicing (9 cases), in-frame insertion (2 cases), microduplication, and null variants (1 case each). Notably, one patient exhibited concurrent in-frame insertion and nonsense variants. Figure 6 illustrates the distribution of *NKX2-1* gene variants and deletions categorized by the respiratory clinical phenotype of the patients. Notably, all patients (*n* = 3) diagnosed with lung cancer exhibit variants classified as nonsense mutations.

### Treatment for *NKX2-1*-RD

Treatment was varied, reflecting the complexity of the respiratory conditions observed. Oxygen supplementation was provided to 76% of patients (66 out of 86), with a mean duration of oxygen therapy (based on 41 patients) lasting 29.4 months. Non-invasive ventilation (NIV) was applied in 8 out of 34 cases (22, 27, 36, 37, 41, 44), while invasive ventilation was employed in 39 out of 70 patients. High-frequency oscillation (HFO) was utilized in 4 out of 48 cases (27, 33, 52, 53), inhaled nitric oxide (iNO) was administered to 5 out of 42 patients (22, 33, 44, 52, 53), and extracorporeal membrane oxygenation (ECMO) was used in 7 out of 82 patients (29, 36, 45, 53).

In terms of pharmaceutical interventions, prescriptions were distributed as follows: Long-term antibiotics were prescribed for 2 out of 33 cases, short-term antibiotics for 16 out of 32 cases, surfactant for 14 out of 47 cases, inhaled corticosteroids (ICS) for 4 out of 21 cases, systemic steroids for 27 out of 46 cases, azithromycin for 18 out of 51 cases, hydroxychloroquine (HCQ) for 10 out of 51 cases, and pirfenidone or nintedanib for 1 out of 29 cases (41).

### *NKX2-1*-RD shows diverse long-term respiratory trajectories

Age at the last visit was available for 73 out of 148 patients (49%), with a mean of 14.8 years, a median of 6.3 years, and a range from 0.1 to 73.0 years. The duration of follow-up was provided for 52 out of 148 patients (35%), with a mean duration of 5.6 years, a median of 3.3 years, and a range from 0.1 to 24 years. The pulmonary status at the last follow-up revealed diverse outcomes among the 71 patients for whom details were available. These outcomes included 16 patients with no respiratory symptoms, 13 with chronic respiratory insufficiency, 13 who succumbed to respiratory failure, 11 with mild symptoms (sick but improved, dyspnea at rest, chronic cough, exercise intolerance), 6 with recurrent infections, 5 with pulmonary fibrosis, and 4 who passed away due to causes unrelated to respiratory insufficiency (such as lung cancer, pulmonary arterial hypertension, etc.). Five patients underwent double lung transplantation (DLTX), with an age range spanning from 7 months to 13 years (29) (Supplementary Data 6). All these patients presented with chronic respiratory insufficiency: 4 had RDS progressing to ILD, and 1 had ILD without neonatal RDS. Additionally, 3 patients experienced recurrent wheezing, asthma, or obstructive lung disease, while information on respiratory status was not available for 77 patients. The overall survival of informed cases was about 60%.

Concerning the group with recurrent infections, age at the last visit was ascertainable for 34 out of 58 patients (59%), demonstrating a mean age of 12.3 years, a median of 5.5 years, and a range spanning from 4 months to 63 years.

**Figure 7:**
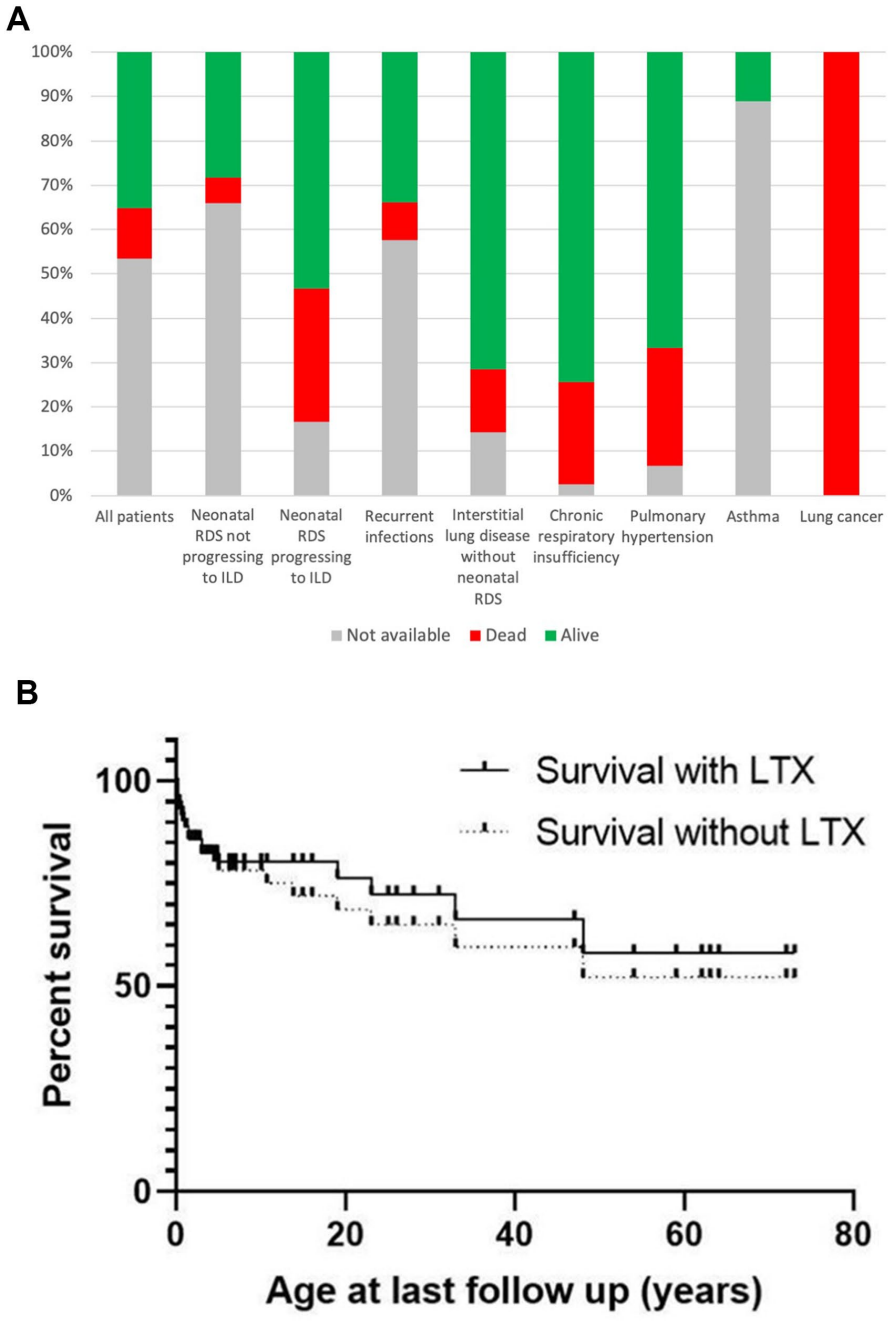
**(A)** Distribution of patients across respiratory phenotypes stratified by vital status. **(B)** Kaplan–Meier survival curve of *NKX2-1* RD patients with respiratory manifestations, comparing those who have undergone lung transplantation (LTX) and those who have not. Note: All LTX occurred before age 20y.

Figure 7A illustrates the distribution of patients across various respiratory phenotypes, stratified by vital status. Figure 7B displays the Kaplan–Meier survival curve of patients with respiratory pathology, comparing those who have undergone lung transplantation (LTX) and those who have not.

### Quality assessment of the included studies

According to the assessment tools used in our systematic review, 79% (*n* = 30) of case series and case reports had poor methodological quality, 18% (*n* = 7) had medium quality, and 3% (*n* = 1) had good quality. The scores obtained for each item, as well as the total score for each reference, are listed in Supplementary Data 7.

## Discussion

This systematic review marks the first extensive examination of evidence concerning respiratory manifestations in *NKX2-1*-RD patients. Those with *NKX2-1*-RD present diverse respiratory conditions, spanning from neonatal respiratory distress to various severe lung diseases, including interstitial lung disease and lung cancer. Significantly, respiratory manifestations stand out as the leading cause of mortality in this patient group (11). An important, often overlooked respiratory problem are recurrent infections, often viral in nature, with consecutive protracted bacterial bronchitis. Such episodes may need aggressive treatment, as in a structurally hypoplastic lung, infections may lead to additional tissue damage and may trigger exacerbations of ILD. For a significant number of patients with end-stage lung disease, only lung transplantation is an option (29). This needs early attention to refer the patients in time to the appropriate transplant centers. The lack of consensus on respiratory follow-up protocols for *NKX2-1* patients prior to this study underscores the necessity of such investigations.

By elucidating the complexities of *NKX2-1*-RD, our research highlights the criticality of evaluating thyroid function in the context of neonatal respiratory distress syndrome (RDS), specifically among females. Our research identifies a sex-specific distribution in *NKX2-1*-RD, with a male-to-female ratio of 1:1.32 in the overall cohort. This pattern is particularly evident in neonatal RDS cases that do not progress to interstitial lung disease (ILD), where females are more frequently affected. In contrast, among patients with neonatal RDS progressing to ILD, males are more prevalent, with a statistically significant difference in sex distribution. Given the known association between male sex and neonatal RDS, a thorough assessment of thyroid function remains critical for prompt diagnosis and intervention in all affected neonates. In addition, we support proactive measures by urging the promotion of genetic testing for mutations causing surfactant dysfunction in neonates who are fully developed and have no other plausible explanations for RDS.

Furthermore, it is crucial to emphasize the significance of conducting a thyroid profile in patients who present with chronic respiratory insufficiency, recurrent infections, interstitial lung disease, or pulmonary hypertension. This recommendation is applicable in situations where neonatal metabolic screening is normal or neonatal RDS is absent.

The identification of risk factors contributing to the progression to ILD in neonates with RDS remains inconclusive in our review. Acknowledging the significance of comprehending the factors influencing ILD development in this vulnerable population, we advocate for an elevated focus in forthcoming research endeavors to unravel these intricacies. As an initiative, an *NKX2-1* patient registry has been established to enable tracking the natural history of patients, which would be beneficial in furthering our understanding of this condition.

In the clinical management of patients with *NKX2-1*-RD, diagnostic modalities, including computed tomography (CT), pulmonary function tests, chest radiography (CR), and lung biopsy, should be utilized with caution. These evaluations are crucial in situations where a more precise phenotypic characterization is required, as they contribute to the tailored indication of treatments and the prediction of outcomes. Nevertheless, it is advisable to exercise caution when considering the routine or preventative use of these tests, especially for those who are asymptomatic. The determination to proceed with CT, pulmonary function tests, and lung biopsy should be predicated on particular clinical indications. This entails verifying that the potential advantages, such as improved diagnostic accuracy and customized therapeutic approaches, surpass the associated risks and burdens of these diagnostic interventions.

Patients undergoing genetic analysis revealed a spectrum of *NKX2-1* gene alterations, encompassing 120 pathogenic variants and 27 pathogenic deletions. Among these, nonsense mutations were identified in 31 cases. Strikingly, all instances of lung cancer were associated with nonsense variants (19, 26, 41). This specific correlation between nonsense mutations in *NKX2-1* and pulmonary malignancy prompts further exploration into potential underlying biological mechanisms, warranting attention in the context of similar associations in the existing literature. This finding could influence the development of targeted screening strategies for this high-risk subset of patients.

This study presents several limitations that should be considered when interpreting its findings. Firstly, the majority of the included studies are case series and case reports, implying inherent limitations in extrapolating the results. Furthermore, the majority of the included studies (70%) have low methodological quality. Additionally, the patient sample is relatively small due to the rarity of *NKX2-1*-RD, potentially impacting the generalizability of the results. The lack of comprehensive data and variable reporting quality contribute to uncertainty in the overall quality of evidence. Moreover, the varying duration of follow-up and absence of control groups limit the ability to make direct comparisons and draw definitive conclusions about the effectiveness of interventions. Heterogeneity in administered treatments and the lack of detailed information on relevant outcomes also pose significant limitations. Finally, the incomplete data on long-term respiratory status for a considerable number of patients hinders a comprehensive understanding of long-term respiratory trajectories in individuals with *NKX2-1*-RD.

The fact that the available evidence came from case series or case reports downgrades the initial quality. Due to the lack of reported data and inadequate study design, it has not been possible to quantitatively estimate the risk of bias, inconsistency, indirectness, imprecision, and publication bias. The indirectness of the study was considered low since the available evidence answered the initial research questions. Otherwise, the imprecision of the results was high due to the small sample size and the lack of reporting of interest results.

In terms of publication bias, conducting an objective assessment was challenging due to the limited availability of data from unpublished studies. The overall quality of the evidence analyzed in this study was considered low due to the previously discussed factors.

This study exhibits several strengths that contribute to its relevance and utility. Firstly, it represents the inaugural comprehensive review of respiratory manifestations in patients with *NKX2-1*-RD, providing a thorough synthesis of the available evidence to date. The incorporation of diverse respiratory manifestations, ranging from neonatal respiratory distress syndrome to severe lung diseases, addresses the breadth of clinical presentation in this population, underscoring the significance of respiratory manifestations as a leading cause of mortality.

### Suggestions for practical insights into *NKX2-1* in the spectrum of respiratory disease

Table 2 delineates the considerations for suspecting *NKX2-1* in patients with various respiratory phenotypes, highlights the distinctions in treatment compared to *NKX2-1*-negative patients with similar respiratory phenotypes, and provides specific key points for each pathological subgroup.

**Table 2:** Clinical and genetic characteristics of patients with *NKX2-1*-RD and associated recommendations.

	Neonatal RDS* not progressing to ILD	Neonatal RDS* progressing to ILD	Recurrent infections	Interstitial lung disease without neonatal RDS	Chronic respiratory insufficiency	Pulmonary hypertension	Asthma	Lung cancer
What distinctions in characteristics can be observed between patients unaffected by *NKX2-1* and those affected by it?	Congenital hypothyroidism. More frequent in females than expected (inverted ratio male: female 1:1,32)	Congenital hypothyroidism.	Chorea and/or congenital hypothyroidism and/or neonatal respiratory distress in the mature or almost mature neonate in the mature or almost mature neonate	Hypothyroidism and neurological symptoms	Chorea and/or congenital hypothyroidism and/or neonatal respiratory distress, or neonatal respiratory distress	Chorea and/or congenital hypothyroidism and/or neonatal respiratory distress	Chorea and/or congenital hypothyroidism and/or neonatal respiratory distress	All three reported patients had nonsense mutations
Are there any distinct recommendations that can be provided for this group of patients?	Thyroid function assessment is mandatory for all patients with neonatal RDS. Genetic testing for surfactant dysfunction mutations in mature neonates without any other explanation of RDS	Thyroid function assessment is mandatory for all patients with neonatal RDS. Genetic testing for surfactant dysfunction mutations in mature neonates without any other explanation of RDS. Follow-up patients at least into childhood	Thyroid function assessment is mandatory for all patients with recurrent infections. Chest x-ray and exercise testing in a healthy state for desaturations.	Thyroid function assessment is mandatory for all patients with interstitial lung disease. Pediatric pneumology workup, including genetic testing for surfactant dysfunction mutations	Thyroid function assessment is mandatory for all patients with chronic respiratory insufficiency. Submit to pediatric pneumology for lung function testing, including exercise, chest imaging, and possibly genetic testing for surfactant dysfunction mutations.	Thyroid function assessment is mandatory for all patients with pulmonary hypertension. Submit to pediatric pneumology for lung function testing, including exercise, chest imaging and possibly genetic testing for surfactant dysfunction and pulmonary hypertension mutations.	If not inhaled steroid responsive, consider pediatric pneumology investigations, including lung function testing, including exercise, chest imaging and possibly genetic testing.	If unexplained respiratory symptoms are evaluated, chest CT should be done in older subjects with *NKX2.1*-RD

^*^This applies to neonates born at term (>37 weeks gestational age), as well as those falling within the category of very preterm or the entire moderate to late preterm group (30–36 weeks), who exhibit respiratory distress without a satisfactory explanation linked to their immaturity.

## Conclusion

In conclusion, this study presents the first comprehensive review of respiratory manifestations in patients with *NKX2-1*-RD, shedding light on the diverse clinical spectrum from neonatal respiratory distress to severe lung diseases, such as interstitial lung disease and lung cancer. The findings underscore the critical role of respiratory manifestations as a primary cause of mortality in this patient cohort. Despite certain limitations, including the predominantly low methodological quality of the reviewed studies, this work lays the foundation for future investigations and emphasizes the need for standardized respiratory follow-up protocols.

### Key findings respiratory disorders in *NKX2-1*-RD

- Critical age of onset: The age at which respiratory symptoms manifest is diverse, with neonatal respiratory distress syndrome being a prominent initial presentation, emphasizing the importance of early detection and intervention.
- Clinical diversity: This study reveals a wide spectrum of respiratory conditions associated with *NKX2-1*-RD, encompassing neonatal respiratory distress to various severe lung diseases, highlighting the heterogeneous nature of this clinical entity.
- Treatment complexity: The multifaceted nature of respiratory conditions in *NKX2-1*-related disorders is reflected in the diverse treatment modalities employed, ranging from oxygen supplementation to advanced interventions like extracorporeal membrane oxygenation (ECMO).
- Leading cause of mortality: Respiratory manifestations emerge as the predominant cause of mortality in patients with *NKX2-1-*RD, underscoring the significance of understanding and managing these respiratory conditions for improved patient outcomes.
- Long-term respiratory trajectories: Long-term follow-up reveals diverse respiratory outcomes, with some patients exhibiting no respiratory symptoms while others experience chronic respiratory insufficiency, recurrent infections, or succumb to respiratory failure.

## Data Availability

All relevant data is contained within the article. The original contributions presented in the study are included in the article/Supplementary material, further inquiries can be directed to the corresponding author/s.
