# Supplementary material for: Respiratory and other organ manifestations in NKX2-1-related disorders: a systematic review
Source: Front Med (Lausanne). 2025 May 6;12:1507513. doi: 10.3389/fmed.2025.1507513 (PMC12090872; doi:10.3389/fmed.2025.1507513)
Supplement: Supplementary file 1 [file Supplementary_file_1.docx]

**Supplementary Data 1. PICOs QUESTION. Lung diseases in NKX2-1-related disorders**

**Generic question:** *What sort of pulmonary follow-up is recommended to monitor the onset of lung diseases in NKX2-1-related disorders?*

| **Structured question 1*:*** *What are the best procedures for diagnosis of lung diseases in patients with NKX2-1-related disorders?* | | |
| --- | --- | --- |
| Criteria | Inclusion criteria | Exclusion criteria |
| **Population** | 1. Patients from all ages with a genetic confirmation of the disease (mutations in *TTF-1/NKX2-1* or deletion in 14q13.3 chromosome) 2. Patients with lung disease including, neonatal respiratory distress syndrome with or without pulmonary hypertension, interstitial lung disease (children´s interstitial lung disease, tachypnea of infancy, neuroendocrine cell hyperplasia of infancy), recurrent pulmonary infection, disruption of pulmonary surfactant, lethal respiratory failure, lung cancer, large cell lung carcinoma, pulmonary alveolar proteinosis, chronic bronchitis recurrent infections, asthma, lung malformation, disrupted lung growth (histology).) | 1. Non-human studies |
| **Intervention** | - Physical examination - Pulse oximetry test - Blood test (blood gases, hemogram, reactive c protein, blood culture, etc.) - Pulmonary function testing - Exercise tolerance testing - Bronchoalveolar lavage - Diffusion capacity for carbon monoxide (DLCO) - Image (Chest X-ray, CT scan, PET-CT scan) - Lung biopsy (bronchoscopy, thoracoscopy, mediastinoscopy, percutaneous needle biopsy, etc.) | None |
| **Comparator** | None | None |
| **Outcome**s | 1. Test results in relation to diagnosis |  |
| **Study design** | Primary studies  Systematic reviews | Narratives reviews  Conference abstracts  Editorials  Letters to the editor |
| **Languages** | All languages | None |

| **Structured question 2**: *What are the best procedures for treatment and follow-up of lung diseases in patients with NKX2-1-related disorders?* | | |
| --- | --- | --- |
| Criteria | Inclusion criteria | Exclusion criteria |
| **Population** | 1. Patients from all ages with a genetic confirmation of the disease (mutations in *TTF-1/NKX2-1* or deletion in 14q13.3 chromosome) 2. Patients with lung disease including, neonatal respiratory distress syndrome with or without pulmonary hypertension, interstitial lung disease (children´s interstitial lung disease, tachypnea of infancy, neuroendocrine cell hyperplasia of infancy), recurrent pulmonary infection, disruption of pulmonary surfactant, lethal respiratory failure, lung cancer, large cell lung carcinoma, pulmonary alveolar proteinosis, chronic bronchitis recurrent infections, asthma, lung malformation, disrupted lung growth (histology). | 1. Non-human studies |
| **Intervention** | 1. Supplementary oxygen 2. Noninvasive and invasive respiratory support 3. Medication (bronchodilators, corticosteroid, immunosuppressive therapy, antibiotics, exogenous surfactant, etc.) 4. Chest physiotherapy 5. Lung transplant 6. Cancer surgery 7. Radiotherapy 8. Chemotherapy 9. Supportive care: nutrition, immunization, environmental care | None |
| **Comparator** | None | None |
| **Outcome**s | Indication for hospitalization  Treatment   1. Effectiveness 2. Adverse effect of treatments 3. Treatment in special situation (pregnancy)   Follow-up   1. Lung outcomes 2. Patient and professional education, quality of life, self-management in adulthood |  |
| **Study design** | Primary studies  Systematic reviews  Previous CPGs | Narratives reviews  Conference abstracts  Editorials  Letters to the editor |
| **Languages** | All languages | None |
