# Supplementary material for: Respiratory and other organ manifestations in NKX2-1-related disorders: a systematic review
Source: Front Med (Lausanne). 2025 May 6;12:1507513. doi: 10.3389/fmed.2025.1507513 (PMC12090872; doi:10.3389/fmed.2025.1507513)
Supplement: Supplementary file 2 [file Supplementary_file_2.docx]

| **Supplementary Data 2** List of final included and excluded articles used in the study and the reasons for the exclusion. |
| --- |
| **INCLUDED STUDIES** |
| 1. Asmus F, Horber V, Pohlenz J , Schwabe D, Zimprich A, Munz M, Schöning M, Gasser T. A novel TITF-1 mutation causes benign hereditary chorea with response to levodopa. Neurology Jun 2005, 64 (11) 1952-1954. DOI: 10.1002/mds.21135. |
| 1. Barnett CP, Mencel JJ, Gecz J, Waters W, Kirwin SM, Vinette KM, et al. Choreoathetosis, congenital hypothyroidism and neonatal respiratory distress syndrome with intact NKX2-1. Am J Med Genet A. 2012;158a(12):3168-73. DOI: 10.1002/ajmg.a.35456. |
| 1. Carré A, Szinnai G, Castanet M, Sura-Trueba S, Tron E, Broutin-L'Hermite I, et al. Five new TTF1/NKX2.1 mutations in brain-lung-thyroid syndrome: rescue by PAX8 synergism in one case. Hum Mol Genet. 2009;18(12):2266-76. DOI: 10.1093/hmg/ddp162. |
| 1. Devos D, Vuillaume, I, De Becdelievre, A, De Martinville, B, Dhaenens, CM, Cuvellier, JC, Cuisset, JM, Vallée, L, Lemaitre, MP, Bourteel, H, Hachulla, E, Wallaert, B, Destée, A, Defebvre, L and Sablonnière, B. New syndromic form of benign hereditary chorea is associated with a deletion of TITF-1 and PAX-9 contiguous genes. Mov. Disord. 2006; 21: 2237-2240. https://doi.org/10.1002/mds.21135. |
| 1. Doyle DA, Gonzalez I, Thomas B, Scavina M. Autosomal dominant transmission of congenital hypothyroidism, neonatal respiratory distress, and ataxia caused by a mutation of NKX2-1. J Pediatr. 2004;145(2):190-3. DOI:10.1016/j.jpeds.2004.04.011. 2. Ferrara AM, De Michele G, Salvatore E, Di Maio L, Zampella E, Capuano S, et al. A novel NKX2.1 mutation in a family with hypothyroidism and benign hereditary chorea. Thyroid. 2008;18(9):1005-9. DOI: 10.1089/thy.2008.0085. |
| 1. Ferrara JM, Adam OR, Kirwin SM, Houghton DJ, Shepherd C, Vinette KM, Litvan I. Brain-lung-thyroid disease: clinical features of a kindred with a novel thyroid transcription factor 1 mutation. J Child Neurol. 2012;27(1):68-73. DOI: 10.1177/0883073811413584. |
| 1. Galambos C, Levy H, Cannon CL, Vargas SO, Reid LM, Cleveland R, Lindeman R, deMello DE, Wert SE, Whitsett JA, Perez-Atayde AR, Kozakewich H. Pulmonary pathology in thyroid transcription factor-1 deficiency syndrome. Am J Respir Crit Care Med. 2010;182(4):549-54. DOI: 10.1164/rccm.201002-0167CR. |
| 1. Gillett ES, Deutsch GH, Bamshad MJ, McAdams RM, Mann PC. Novel NKX2.1 mutation associated with hypothyroidism and lethal respiratory failure in a full-term neonate. J Perinatol. 2013;33(2):157-60. DOI: 10.1038/jp.2012.50. 2. Glik A, Vuillaume I, Devos D and Inzelberg R. Psychosis, short stature in benign hereditary chorea: A novel thyroid transcription factor-1 mutation. Mov. Disord. 2008; 23: 1744-1747. https://doi.org/10.1002/mds.22215. |
| 1. Gras D, Jonard L, Roze E, Chantot-Bastaraud S, Koht J, Motte J, et al. Benign hereditary chorea: phenotype, prognosis, therapeutic outcome and long term follow-up in a large series with new mutations in the TITF1/NKX2-1 gene. J Neurol Neurosurg Psychiatry. 2012;83(10):956-62. https://dx.doi.org/10.1136/jnnp-2012-302505. |
| 1. Gu R, Ye G, Zhou Y, Jiang Z. Combined mutations of NKX2-1 and SFTPC genes for refractory low oxyhemoglobin saturation and interstitial pneumonia: A case report. Medicine 2020;99:12(e19650). https://dx.doi.org/10.1097/MD.0000000000019650 |
| 1. Guillot L, Carré A, Szinnai G, et al. NKX2-1 mutations leading to surfactant protein promoter dysregulation cause interstitial lung disease in "Brain-Lung-Thyroid Syndrome". Human Mutation. 2010;31(2):E1146-62. DOI: 10.1002/humu.21183. PMID: 20020530. |
| 1. Hamvas A, Deterding RR, Wert SE, et al. Heterogeneous pulmonary phenotypes associated with mutations in the thyroid transcription factor gene NKX2-1 Chest. 2013;144 (3):794-804. https://dx.doi.org/10.1378/chest.12-2502. |
| 1. Hanes I, Mestre T, Doja A. An 8-year-old boy with ataxia and abnormal movements. Paediatr Child Health. 2019;24(5):297-298. DOI: 10.1093/pch/pxy178. |
| 1. Hu X, Liu J, Guo R, Guo J, Zhao Z, Li W, Xu B, Hao C. A novel 14q13.1-21.1 deletion identified by CNV-Seq in a patient with brain-lung-thyroid syndrome, tooth agenesis and immunodeficiency. Mol Cytogenet. 2019;12:51. doi: 10.1186/s13039-019-0463-z. 2. Iwatani N, Mabe H, Devriendt K, Kodama M, Miike T. Deletion of NKX2.1 gene encoding thyroid transcription factor-1 in two siblings with hypothyroidism and respiratory failure. J Pediatr. 2000;137(2):272-6. DOI: 10.1067/mpd.2000.107111. 3. Jovien S, Borie R, Doummar D, Clement A, Nathan N. Respiratory Distress, Congenital Hypothyroidism and Hypotonia in a Newborn. Respiration 2016; 92(3): 188–191. https://doi.org/10.1159/000449136 4. Kleinlein B, Griese M, Liebisch G, Krude H, Lohse P, Aslanidis C, et al. Fatal neonatal respiratory failure in an infant with congenital hypothyroidism due to haploinsufficiency of the NKX2-1 gene: alteration of pulmonary surfactant homeostasis. Arch Dis Child Fetal Neonatal Ed. 2011;96(6):F453-6. https://dx.doi.org/10.1136/adc.2009.180448. |
| 1. Koht J, Løstegaard SO, Wedding I, Vidailhet M, Louha M, Tallaksen CM. Benign hereditary chorea, not only chorea: a family case presentation. Cerebellum Ataxias. 2016;3:3. DOI: 10.1186/s40673-016-0041-7. |
| 1. LeMoine BD, Browne LP, Weinman JP, Liptzin DR, Deterding RR, Galambos C. High-resolution computed tomography findings of thyroid transcription factor 1 deficiency (NKX2–1 mutations). Pediatric Radiology 2019; 49(7), 869-875. https://dx.doi.org/10.1007/s00247-019-04388-3. |
| 1. Lynn MM, Simon D, Kasi AS. Hypoxaemia and interstitial lung disease in an infant with hypothyroidism and hypotonia. BMJ Case Rep. 2020;13(12). DOI: 10.1136/bcr-2020-238466. |
| 1. Maquet E, Costagliola S, Parma J, Christophe-Hobertus C, Oligny LL, Fournet JC, et al. Lethal respiratory failure and mild primary hypothyroidism in a term girl with a de novo heterozygous mutation in the TITF1/NKX2.1 gene. J Clin Endocrinol Metab. 2009;94(1):197-203. https://dx.doi.org/10.1210/jc.2008-1402. |
| 1. Marić N, Ljuboja O. Pulmonary Hemorrhage in a Patient with Brain-Lung-Thyroid Syndrome Caused by a p.T86fs Variant in the NKX2-1 Gene. Central Eur J Paed 2020;16(2):155-160. DOI:10.5457/p2005-114.271. |
| 1. Mirza A, Martinez M, Kilaikode S. Unusual Cause of Respiratory Distress in a Term Neonate. Ochsner Journal 2022;22(2):196-198. DOI: 10.31486/toj.21.0101. |
| 1. Nattes E, Lejeune S, Carsin A, Borie R, Gibertini I, Balinotti J, et al. Heterogeneity of lung disease associated with NK2 homeobox 1 mutations. Respir Med. 2017;129:16-23. https://dx.doi.org/10.1016/j.rmed.2017.05.014. 2. Nevel RJ, Garnett ET, Worrell JA, Morton RL, Nogee LM, Blackwell TS, Young LR. Persistent Lung Disease in Adults with NKX2.1 Mutation and Familial Neuroendocrine Cell Hyperplasia of Infancy. Ann Am Thorac Soc. 2016;13(8):1299-304. DOI: 10.1513/AnnalsATS.201603-155BC. |
| 1. Parnes M, Bashir H, Jankovic J. Is Benign Hereditary Chorea Really Benign? Brain-Lung-Thyroid Syndrome Caused by NKX2-1 Mutations. Mov Disord Clin Pract. 2019;6(1):34-9. DOI: 10.1002/mdc3.12690. |
| 1. Peall KJ, Lumsden D, Kneen R, Madhu R, Peake D, Gibbon F, et al. Benign hereditary chorea related to NKX2.1: expansion of the genotypic and phenotypic spectrum. Dev Med Child Neurol. 2014; 56(7):642-8. doi: 10.1111/dmcn.12323. 2. Pohlenz J, Dumitrescu A, Zundel D, Martiné U, Schönberger W, Koo E et al. Partial deficiency of Thyroid transcription factor 1 produces predominantly neurological defects in humans and mice. J. Clin. Invest. 2002; 109:469–473. DOI:10.1172/JCI200214192. |
| 1. Prasad R, Nicholas AK, Schoenmakers N, Barton J. Haploinsufficiency of NKX2-1 in Brain-Lung-Thyroid Syndrome with Additional Multiple Pituitary Dysfunction. Horm Res Paediatr. 2019;92(5):340-344. doi: 10.1159/000503683. 2. Safi KH, Bernat JA, Keegan CE, Ahmad A, Hershenson MB, Arteta M. Interstitial lung disease of infancy caused by a new NKX2-1 mutation. Clin Case Rep. 2017;5(6):739-743. doi: 10.1002/ccr3.901. |
| 1. Salerno T, Peca D, Menchini L, Schiavino A, Petreschi F, Occasi F, et al. Respiratory insufficiency in a newborn with congenital hypothyroidism due to a new mutation of TTF-1/NKX2.1 gene. Pediatr Pulmonol. 2014;49(3):E42-4. https://dx.doi.org/10.1002/ppul.22788. |
| 1. Salvado M, Boronat-Guerrero S, Hernández-Vara J, Álvarez-Sabin J. [Chorea due to TITF1/NKX2-1 mutation: phenotypical description and therapeutic response in a family]. Rev Neurol. 2013;56(10):515-20. |
| 1. Sutton RM, Bittar HT, Sullivan DI, Silva AG, Bahudhanapati H, Parikh AH, Zhang Y, Gibson K, McDyer JF, Kass DJ, Alder JK. Rare surfactant-related variants in familial and sporadic pulmonary fibrosis. Hum Mutat. 2022;43(12):2091-2101. DOI: 10.1002/humu.24476. |
| 1. Thorwarth A, Schnittert-Hübener S, Schrumpf P, Müller I Jyrch S, Dame C et al. Comprehensive genotyping and clinical characterisation reveal 27 novel NKX2-1 mutations and expand the phenotypic spectrum. Journal of Medical Genetics 2014; 51:375-387. DOI:10.1136/jmedgenet-2013-102248. |
| 1. Villamil-Osorio M, Yunis LK, Quintero L, Restrepo-Gualteros S, Yunis JJ, Jaramillo L, et al. [Brain-lung-thyroid syndrome in a newborn with deletion 14q12-q21.1]. Andes Pediatr. 2021;92(6):930-6. https://dx.doi.org/10.32641/andespediatr.v92i6.3287. |
| 1. Willemsen MA, Breedveld GJ, Wouda S, Otten BJ, Yntema JL, Lammens M, de Vries BB. Brain-Thyroid-Lung syndrome: a patient with a severe multi-system disorder due to a de novo mutation in the thyroid transcription factor 1 gene. Eur J Pediatr. 2005;164(1):28-30. doi: 10.1007/s00431-004-1559-x. |

**EXCLUDED STUDIES**

| **Wrong patient population** |
| --- |
| 1. Balicza P, Grosz Z, Molnár V, Illés A, Csabán D, Gézsi A et al. NKX2-1 New Mutation Associated With Myoclonus, Dystonia, and Pituitary Involvement. Front. Genet. 2018;9:335. DOI: 10.3389/fgene.2018.00335. 2. Breedveld GJ, van Dongen JWF, Danesino C, Guala A, Percy AK, Dure LS et al. Mutations in TITF-1 are associated with benign hereditary chorea. Human molecular genetics 2002;11(8):971-9. 3. Canals J, Navarro A, Vila C, Canals JM, Díaz T, Acosta-Plasencia M et al. Human embryonic mesenchymal lung-conditioned medium promotes differentiation to myofibroblast and loss of stemness phenotype in lung adenocarcinoma cell lines. J Exp Clin Cancer Res 2022;41, 37. https://doi.org/10.1186/s13046-021-02206-z. 4. Davies SJ, Gosney JR, Hansell DM, Wells AU, du Bois RM, Burke MM et al. Diffuse idiopathic pulmonary neuroendocrine cell hyperplasia: an under-recognised spectrum of disease. Thorax. 2007 Mar;62(3):248-52. DOI: 10.1136/thx.2006.063065. 5. Eldridge WB, Zhang Q, Faro A, Sweet SC, Eghtesady P, Hamvas A et al. Outcomes of Lung Transplantation for Infants and Children with Genetic Disorders of Surfactant Metabolism. J Pediatr. 2017 May;184:157-164.e2. DOI: 10.1016/j.jpeds.2017.01.017. 6. Escudero AG, Zarco ER, Arjona JC, Moreno MJ, Rodríguez KG, Benítez AV, et al. Expression of developing neural transcription factors in diffuse idiopathic pulmonary neuroendocrine cell hyperplasia (DIPNECH). Virchows Arch. 2016 Sep;469(3):357-63. DOI: 10.1007/s00428-016-1962-5. 7. Griese M, Lorenz E, Hengst M, Schams A, Wesselak T, Rauch D et al. Surfactant proteins in pediatric interstitial lung disease. Pediatr Res 2016;79,34–41. https://doi.org/10.1038/pr.2015.173. 8. Hanes I, Mestre T, Doja A. An 8-year-old boy with ataxia and abnormal movements. Paediatr Child Health. 2019 Aug;24(5):297-298. DOI: 10.1093/pch/pxy178. 9. Kharbanda M, Hermanns P, Jones J, Pohlenz J, Horrocks I, Donaldson M. A further case of brain-lung-thyroid syndrome with deletion proximal to NKX2-1. Eur J Med Genet. 2017 May;60(5):257-260. DOI: 10.1016/j.ejmg.2017.03.001. 10. Konishi T, Kono S, Fujimoto M, Terada T, Matsushita K, Ouchi Y et al. Benign hereditary chorea: dopaminergic brain imaging in patients with a novel intronic NKX2.1 gene mutation. J Neurol. 2013 Jan;260(1):207-13. DOI: 10.1007/s00415-012-6618-z. 11. Peca D, Petrini S, Tzialla C, Boldrini R, Morini F, Stronati M et al. Altered surfactant homeostasis and recurrent respiratory failure secondary to TTF-1 nuclear targeting defect. Respir Res. 2011 Aug 25;12(1):115. DOI: 10.1186/1465-9921-12-115. 12. Uematsu M, Haginoya K, Kikuchi A, Nakayama T, Kakisaka Y, Numata Y et al. Hypoperfusion in caudate nuclei in patients with brain-lung-thyroid syndrome. J Neurol Sci. 2012 Apr 15;315(1-2):77-81. DOI: 10.1016/j.jns.2011.11.025. 13. Williamson S, Kirkpatrick M, Greene S, Goudie D. A Novel Mutation of NKX2-1 Affecting 2 Generations With Hypothyroidism and Choreoathetosis: Part of the Spectrum of Brain-Thyroid-Lung Syndrome. Journal of Child Neurology 2014, Vol. 29(5) 666-669. DOI: 10.1177/0883073813518243. |
| **Wrong outcomes** |
| 1. de Filippis T, Marelli F, Vigone MC, Di Frenna M, Weber G, Persani L. Novel NKX2-1 Frameshift Mutations in Patients with Atypical Phenotypes of the Brain-Lung-Thyroid Syndrome. Eur Thyroid J. 2014 Dec;3(4):227-33. DOI: 10.1159/000366274. 2. Hermanns P, Kumorowicz-Czoch M, Grasberger H, Refetoff S, Pohlenz J. Novel Mutations in the NKX2.1 gene and the PAX8 gene in a Boy with Brain-Lung-Thyroid Syndrome. Exp Clin Endocrinol Diabetes. 2018 Feb;126(2):85-90. DOI: 10.1055/s-0043-119875. 3. Strickler A, Boza ML, Koppmann A, Gonzalez S. Autoimmune pulmonary proteinosis in a Chilean teenager, a rare aetiology of interstitial lung disease. BMJ Case Rep. 2014 May 23;2014:bcr2012006987. DOI: 10.1136/bcr-2012-006987. 4. Moya CM, Zaballos MA, Garzón L, Luna C, Simón R, Yaffe MB, Gallego E, Santisteban P, Moreno JC. TAZ/WWTR1 Mediates the Pulmonary Effects of NKX2-1 Mutations in Brain-Lung-Thyroid Syndrome. J Clin Endocrinol Metab. 2018 Mar 1;103(3):839-852. DOI: 10.1210/jc.2017-01241.  \| **Non-available text** \| \| --- \| \| 1. Fan LL. Why and how is ILD in children different from adults? Respirology 2010;15():10. DOI: 10.1111/j.1400-1843.2010.01863.x. \| |

| **Wrong study design** |
| --- |
| 1. Auyeung V, Planer BC, Chartoff A, Oundjian N. The link between respiratory failure and congenital hypothyroidism. Thyroid 2011;21():A52-A53. https://doi.org/10.1089/thy.2011.2110.abs. 2. Bush A. Management and prognosis. Pediatric Pulmonology 2019;54():S24-S25. DOI: 10.1002/ppul.24371. 3. Bush A, Pabary R. Pulmonary alveolar proteinosis in children. Breathe Jun 2020, 16 (2) 200001; DOI: 10.1183/20734735.0001-2020. 4. Cavanna AE. Behavioural and cognitive dysfunction across basal ganglia disorders. Journal of Neurology, Neurosurgery and Psychiatry. 2012 Oct;83(10):950-951. DOI: 10.1136/jnnp-2012-302834. 5. Chakraborty M, Kotecha S. Pulmonary surfactant in newborn infants and children. Breathe Dec 2013, 9 (6) 476-488; DOI: 10.1183/20734735.006513. 6. Chapman SJ, Wise MP. Idiopathic pulmonary fibrosis: a paradigm of late-onset, single-gene human disease?BMJ Open Respiratory Research 2014;1:e000070. DOI: 10.1136/bmjresp-2014-000070. 7. Chiş AF, Man MA, Chiş BA, Pop CM. Alveolar proteinosis - an underdiagnosed condition in young people. Med Pharm Rep. 2021 Aug;94(Suppl No 1):S40-S42. doi: 10.15386/mpr-2227. 8. Cho K, Ikeda M, Onda T, Furuse Y, Ando A, Nakamura Y. Advances in genetics. Pediatric Pulmonology 2019;54():S22-S23. 9. Coetzee M, De Witt TW. An approach to diagnosing and managing diffuse lung disease presenting in the newborn period. South African Journal of Child Health 2019;13(3):145-150. DOI:10.7196/SAJCH.2019.v13i3.1594. 10. Danhaive O, Peca D, Cutrera R. Role of surfactant protein-B, surfactant protein-C and ATP-binding cassette transporter A3 mutations in infants with surfactant homeostasis disruption and progressive diffuse lung disease. Neonatology 2011;99(4):371. DOI: 10.1159/000326687. 11. Delestrain C, Flamein F, Jonard L, Couderc R, Guillot L, Fanen P, Epaud R. Pathologies respiratoires de l'enfant associées à des anomalies héréditaires du métabolisme du surfactant [Lung diseases in children associated with inherited disorders of surfactant metabolism]. Rev Pneumol Clin. 2013 Aug;69(4):183-9. French. DOI: 10.1016/j.pneumo.2013.05.002. 12. Epaud R, Feldmann D, Guillot L, Clément A. Pathologies respiratoires associées à des anomalies héréditaires du métabolisme du surfactant [Lung diseases associated with inherited disorders of surfactant metabolism]. Arch Pediatr. 2008 Oct;15(10):1560-7. French. DOI: 10.1016/j.arcped.2008.07.016. 13. Epaud R, Jonard L, Ducou-le-Pointe H, Delestrain C, Fanen P, Guillot L, Flamein F. Pathologies génétiques du surfactant [Genetic disorders of surfactant]. Arch Pediatr. 2012 Feb;19(2):212-9. French. DOI: 10.1016/j.arcped.2011.12.004. 14. Ferreira SH, Pereia S, Jacob S, Abreu M, Gonçalves D, Sampaio M et al. Congenital hypothyroidism and Brain-Lung-Thyroid syndrome. Excellence in Pediatrics (2017) 9th Excellence in Pediatrics Conference - 2017 Book of Abstracts, Cogent Medicine, 4:1, ID: 281 / PO1: 20. DOI: 10.1080/2331205X.2017.1408251. 15. Griese M. Chronic interstitial lung disease in children. Eur Respir Rev. 2018 Feb 7;27(147):170100. DOI: 10.1183/16000617.0100-2017. 16. Griese, M; Witt, S; Seidl, E; Kaur, M; Eismann, C; Kappler, M et al. Early onset and familiar Interstitial lung disease: Diagnose molecularly and systematically explore treatment with hydroxychloroquine. Pneumologie 2019;73(SUPPPL. 1). DOI: 10.1055/s-0039-1678090. 17. Guan L, Zhao X, Tang L, Chen J, Zhao J, Guo M et al. Thyroid Transcription Factor-1: Structure, Expression, Function and Its Relationship with Disease. Biomed Res Int. 2021 Sep 28;2021:9957209. DOI: 10.1155/2021/9957209. 18. Gupta A, Zheng SL. Genetic disorders of surfactant protein dysfunction: when to consider and how to investigate Archives of Disease in Childhood 2017;102:84-90. http://dx.doi.org/10.1136/archdischild-2012-303143. 19. Gursoy TR, Onay ZR, Asfuroglu P, Eyüboglu TS, Aslan AT. Clinical features of surfactant metabolism disorders in children. European Respiratory Journal Sep 2020, 56 (suppl 64) 3528; DOI: 10.1183/13993003.congress-2020.3528 20. Hamvas A. Evaluation and management of inherited disorders of surfactant metabolism. Chin Med J (Engl). 2010 Oct;123(20):2943-7. 21. Haubenberger D, Bauer P, Lieba-Samal D, Zimprich A, Auff E, Pirker W. A novel NKX2A-mutation causing benign hereditary chorea is associated with non-progressive striatal D2-receptor dysfunction. Movement Disorders 2012;27():S327. DOI: 10.1002/mds.25051. 22. Hicks J, Mierau G, Wartchow E, Langston C. TTF-1 associated surfactant deficiency: Role of ultrastructural evaluation. Laboratory Investigation 2013;93():504A. DOI: 10.1038/labinvest.2013.37. 23. Inzelberg R, Weinberger M, Gak E. Benign hereditary chorea: an update. Parkinsonism Relat Disord. 2011 Jun;17(5):301-7. DOI: 10.1016/j.parkreldis.2011.01.002. 24. Jo HS. Genetic risk factors associated with respiratory distress syndrome. Korean J Pediatr. 2014 Apr;57(4):157-63. DOI: 10.3345/kjp.2014.57.4.157. 25. Kaler M, Martinez M, Kilaikode S. Unusual cause of respiratory distress in a newborn. Journal of Investigative Medicine 2020;68(2):601. DOI: 10.1136/jim-2020-SRM.414. 26. Kuo CS, Young LR. Interstitial lung disease in children. Curr Opin Pediatr. 2014 Jun;26(3):320-7. doi: 10.1097/MOP.0000000000000094. 27. Limon MA, Parveen S, Rehman A, Jakoby M. Abstract #1001910: Brain-Lung-Thyroid Syndrome (BTLS) Diagnosed in an Adult and Caused by a Newly Recognized Pathogenic Variant of Thyroid Transcription Factor-1 (TTF-1). Endocrine Practice 2021;27(6):S157. DOI: 10.1016/j.eprac.2021.04.799. 28. Mandell E, Kinsella JP, Abman SH. Persistent pulmonary hypertension of the newborn. Pediatr Pulmonol. 2021 Mar;56(3):661-669. DOI: 10.1002/ppul.25073. 29. Mehanna R, Jankovic J. Respiratory problems in neurologic movement disorders. Parkinsonism & related disorders 2010;16(10):628-638. DOI: 10.1016/j.parkreldis.2010.07.004. 30. Moya CM, Zaballos MA, Garzón L, Luna C, Simón R, Yaffe MB, Gallego E, Santisteban P, Moreno JC. TAZ/WWTR1 Mediates the Pulmonary Effects of NKX2-1 Mutations in Brain-Lung-Thyroid Syndrome. J Clin Endocrinol Metab. 2018 Mar 1;103(3):839-852. DOI: 10.1210/jc.2017-01241. 31. Nathan N, Borensztajn K, Clement A. Genetic causes and clinical management of pediatric interstitial lung diseases. Curr Opin Pulm Med. 2018 May;24(3):253-259. DOI: 10.1097/MCP.0000000000000471. 32. Nathan N, Berdah L, Delestrain C, Sileo C, Clement A. Interstitial lung diseases in children. Presse Med. 2020 Jun;49(2):103909. DOI: 10.1016/j.lpm.2019.06.007. 33. NCT04532346. Safety and Efficacy of Hydroxychloroquine in Children's Interstitial Lung Diseases With Genetic Causes: a Randomized Controlled Study. 34. Nettore IC, Mirra P, Ferrara AM, Sibilio A, Pagliara V, Kay CS, Lorenzoni PJ, Werneck LC, Bruck I, Dos Santos LH, Beguinot F, Salvatore D, Ungaro P, Fenzi G, Scola RH, Macchia PE. Identification and functional characterization of a novel mutation in the NKX2-1 gene: comparison with the data in the literature. Thyroid. 2013 Jun;23(6):675-82. DOI: 10.1089/thy.2012.0267. 35. Nogee LM. Genetic causes of surfactant protein abnormalities. Curr Opin Pediatr. 2019 Jun;31(3):330-339. doi: 10.1097/MOP.0000000000000751. 36. Nogee LM. Genetic Surfactant Deficiency Disease in the Newborn. Pediatric Pulmonology 2022;57():S52-S54. DOI: 10.1002/ppul.25961. 37. Reem I, Jacqueline S, Bishay Lara C. Pulmonary hypertension in a limping child: a case of urgen-c. Pulmonary Circulation 2022;12(2): A019. DOI: 10.1002/pul2.12065. 38. Singh J, Jaffe A, Schultz A, Selvadurai H. Surfactant protein disorders in childhood interstitial lung disease. Eur J Pediatr. 2021 Sep;180(9):2711-2721. doi: 10.1007/s00431-021-04066-3. 39. Tamai N, Koinuma G, Takase M. Childhood interstitial lung disease in immunocompetent children in Japan: 9 years' experience. Pediatric Pulmonology 2019;54():S96-S97. DOI: 10.1002/ppul.24373. |

**Wrong publication type**

| 1. Barbian ME, Piazza A, Gauthier T, Williams H. Use of genetic sequencing in premature infant with persistent respiratory failure. Journal of Investigative Medicine 2018; Vol. 66 (2):511-511. Meeting Abstract 394. DOI: 10.1136/jim-2017-000697.394. 2. Borie R, Kannengiesser C, Amselem S, Brugiere O, Bouvry D, Clement A et al. European Respiratory Journal Sep 2018, 52 (suppl 62) PA2233; DOI: 10.1183/13993003.congress-2018.PA2233 3. Cheng P, Li D, Goldfarb SB, Hakonarson H. Genomic Susceptibility to the Development of Pediatric Interstitial Lung Disease. Am J Respir Crit Care Med 2019;199:A4515. DOI: 10.1164/ajrccm-conference.2019.199.1_MeetingAbstracts.A4515. 4. Coghlan MA, Huang HJ, Shifren A, Russell TD, Mohamed S, Zhang Q et al. Rare Mutations Are Over-Represented In Idiopathic Pulmonary Fibrosis. Am J Respir Crit Care Med 2012; 185: A5165. https://doi.org/10.1164/ajrccm-conference.2012.185.1_MeetingAbstracts.A5165 5. Concepcion E, Deutsch G, Lee H. Subpleural cysts on chest tomography in seven months old baby with tachypnea. Am J Respir Crit Care Med 2017;195:A6131. 6. Deterding RR, Dishop M, Uchida DA, Stephan M, Williames L, Lebel RR et al. Thyroid transcription factor 1 gene abnormalities: An under recognized cause of children's interstitial lung disease. Am J Respir Crit Care Med 2010;181:A6725. https://doi.org/10.1164/ajrccm-conference.2010.181.1_MeetingAbstracts.A6725. 7. Epaud R, Kron C, Flamein F, Nimal D, Nathan N, Corvol H et al. Azithromycin in interstitial lung disease associated with surfactant metabolism disorders. Am J Respir Crit Care Med 2010;181:A3993. https://doi.org/10.1164/ajrccm-conference.2010.181.1_MeetingAbstracts.A3993 8. Foster E, Manzar S. Respiratory Distress and Abnormal CT Scan in a Newborn Infant. Neonatal Intensive Care 2022;35(1):69-70. 9. Gallagher RC, Halbower AC, Baker CD, Thomas JA, Saenz M, Goodman SI et al. NKX2.1 haploinsufficiency: A novel etiology of 3-methylglutaconic aciduria associated with chorea. Molecular Genetics and Metabolism 2009;98(1-2):99-100. 10. LeMoine BD, Browne LP, Liptzin DR, Deterding RR, Galambos C, Weinman JP. High-resolution computed tomography findings of thyroid transcription factor 1 deficiency (NKX2-1 mutations). Pediatr Radiol. 2019 Jun;49(7):869-875. DOI: 10.1007/s00247-019-04388-3. 11. Le Pointe HD. Top ten lesions of the pediatric lung. Pediatric Radiology 2012;42():S420-S421. DOI: 10.1007/s00247-012-2383-5. 12. Nathan N, Borie R, Jovien S, Doummar D, Louha M, Beucher J et al. Phenotype heterogeneity in a familial “brain lung thyroid syndrome” related to a novel NKX-2.1 mutation. European Respiratory Journal Sep 2016, 48 (suppl 60) PA3889; DOI: 10.1183/13993003.congress-2016.PA3889. 13. Renoux MC, Serre I, Cambonie G, Rizcallah E, Saguintaah M, Counil FP. Surfactant Protein Deficiency: A Case Of Lung Disease Associated With Hypothyroidism. Am J Respir Crit Care Med 2012; 185:A3373. https://doi.org/10.1164/ajrccm-conference.2012.185.1_MeetingAbstracts.A3373. 14. Young LR, Deutsch GH, Bokulic RE, Brody AS, Nogee LM. A mutation in TTF1/NKX2.1 is associated with familial neuroendocrine cell hyperplasia of infancy. Chest. 2013 Oct;144(4):1199-1206. DOI: 10.1378/chest.13-0811. 15. Young L, Nevel R, Casey A, Fishman M, Welsh S, Liptzin D et al. The national registry for childhood interstitial and diffuse lung diseases: An initial report from the child research network. European Respiratory Journal 2018 52: OA3786; DOI: 10.1183/13993003.congress-2018.OA3786. |
| --- |
| **Wrong language** |
| 1. Chen JH, Zheng YJ. [Brain-lung-thyroid syndrome report of two cases]. Zhonghua Er Ke Za Zhi. 2020 Oct 2;58(10):847-849. Chinese. DOI: 10.3760/cma.j.cn112140-20200118-00039. 2. Kojima Y, Atobe M, Aoki Y, Suzuki M, Itomi K et al. A case of brain-lung-thyroid syndrome showing atypical symptoms. NO TO HATTATSU 2021, Vol.53, Issue 1, Pages 44-48. https://doi.org/10.11251/ojjscn.53.44 |
| 1. Liang R, Ou S, Ding Y, Liu C. A case of brain-lung-thyroid syndrome. Zhong Nan Da Xue Xue Bao Yi Xue Ban. 2022 Mar 28;47(3):396-400. English, Chinese. DOI: 10.11817/j.issn.1672-7347.2022.200998. 2. Zhestkova MA, Ovsyannikov D,Vasilieva TG, Donin IM, Klyukhina YB, Kolmykova AV et al. Brain–lung–thyroid syndrome: Literature review and series of clinical observations. Pediatria. Journal named after G.N. Speransky. 2019; 98. 85-93. Russian. DOI: 10.24110/0031-403X-2019-98-5-85-93. |
