# Supplementary material for: Respiratory and other organ manifestations in NKX2-1-related disorders: a systematic review
Source: Front Med (Lausanne). 2025 May 6;12:1507513. doi: 10.3389/fmed.2025.1507513 (PMC12090872; doi:10.3389/fmed.2025.1507513)
Supplement: Supplementary file 3 [file Supplementary_file_3.docx]

**Supplementary Data 3.** Characteristics of the included studies.

| **Study** | **Country** | **PICO question** | **Study type** | **Study target population** |
| --- | --- | --- | --- | --- |
| **Asmus, 2005** | Germany | Diagnosis | Case report | Family pedigree with NKX2-1 gene mutations |
| **Barnett, 2012** | Australia | Treatment | Case report | - |
| **Carré, 2009** | France | Diagnosis | Case series | Patients with NKX2-1 gene mutations |
| **Devos, 2006** | France | Diagnosis | Case series | Family pedigree with NKX2-1 gene mutations |
| **Doyle, 2004** | USA | Diagnosis/ Treatment | Case series | Family pedigree with dominant transmission of NKX2-1 mutation |
| **Ferrara, 2008** | USA | Diagnosis | Case report | - |
| **Ferrara, 2012** | USA | Diagnosis/ Treatment | Case series | Family pedigree with NKX2-1 gene mutations |
| **Galambos, 2010** | USA | Diagnosis | Case report | - |
| **Gillett, 2013** | USA | Diagnosis | Case report | - |
| **Glik, 2008** | Israel | Diagnosis | Case report | - |
| **Gras, 2012** | France | Diagnosis | Case series | Patients with NKX2-1 gene mutations |
| **Gu, 2020** | China | Treatment | Case report | - |
| **Guillot, 2010** | France | Diagnosis | Case series | Patients with NKX2-1 gene mutations |
| **Hamvas, 2013** | USA | Diagnosis/ Treatment | Case series | Patients with NKX2-1 gene mutations |
| **Hanes, 2018** | Canada | Treatment | Case report | - |
| **Hu, 2019** | China | Treatment | Case report | - |
| **Iwatani, 2000** | Belgium | Diagnosis | Case report | Siblings with a NKX2-1 gene deletion |
| **Jovien, 2016** | France | Diagnosis/ Treatment | Case series | Family pedigree with NKX2-1 gene mutations |
| **Kleinlein, 2010** | Germany | Diagnosis/ Treatment | Case report | - |
| **Koht, 2016** | Norway | Treatment | Case series | Family pedigree with NKX2-1 gene mutations |
| **LeMoine, 2019** | USA | Diagnosis/ Treatment | Case series | 6 children with NKX2-1 mutations |
| **Lynn, 2020** | USA | Diagnosis/ Treatment | Case report | - |
| **Maquet, 2009** | Canada | Diagnosis/ Treatment | Case report | - |
| **Maric, 2020** | Bosnia and Herzegovina | Treatment | Case report | - |
| **Mirza, 2022** | USA | Diagnosis/ Treatment | Case report | - |
| **Nattes, 2017** | France | Diagnosis/ Treatment | Case series | Patients with NKX2-1 gene mutations |
| **Nevel, 2016** | USA | Diagnosis | Case series | Family pedigree with NKX2-1 gene mutations |
| **Parnes, 2019** | USA | Treatment | Case series | Patients with NKX2-1 gene mutations |
| **Peall, 2014** | UK | Diagnosis | Case series | Patients with NKX2-1 gene mutations |
| **Pohlenz, 2002** | USA | Diagnosis | Case report | - |
| **Prasad, 2019** | UK | Diagnosis | Case report | - |
| **Safi, 2017** | USA | Treatment | Case series | Family pedigree with NKX2-1 gene mutations |
| **Salerno, 2014** | Italy | Diagnosis/ Treatment | Case report | - |
| **Salvado, 2013** | Spain | Diagnosis | Case series | Family pedigree with NKX2-1 gene mutations |
| **Sutton, 2022** | USA | Diagnosis | Case series | Patients with NKX2-1 gene mutations |
| **Thorwarth, 2014** | Germany | Diagnosis | Case series | Patients with NKX2-1 gene mutations |
| **Villamil-Osorio, 2021** | Colombia | Diagnosis/ Treatment | Case report | - |
| **Willemsen, 2005** | Netherlands | Diagnosis | Case report | - |
