# Supplementary material for: Respiratory and other organ manifestations in NKX2-1-related disorders: a systematic review
Source: Front Med (Lausanne). 2025 May 6;12:1507513. doi: 10.3389/fmed.2025.1507513 (PMC12090872; doi:10.3389/fmed.2025.1507513)
Supplement: Supplementary file 4 [file Supplementary_file_4.docx]

**Supplementary Data 4:** Summary of patient and reference information for *NKX2-1-*Related Disorders, including genotype, age at onset of pulmonary manifestations, initial pulmonary symptoms, X-ray or CT diagnosis, other diagnostic procedures, lung biopsy, treatment, and brief follow-up details at the patient level.

**Lung cancer**

| **Patient and reference** | **Genotype** | **Number** | **Age at pulmonary manifestations** | **First pulmonary symptoms** | **X-ray or CT diagnosis** | **Other diagnosis procedure** | **Lung biopsy** | **Treatment** | **Follow-up** |
| --- | --- | --- | --- | --- | --- | --- | --- | --- | --- |
| Gras_2012_P3 | c.257dupA | 28 | NA | lung cancer | NA | NA | NA | NA | NA |
| Willemsen_2005_P1 | c.859-860insC | 139 | Neonatal (2 weeks) | neonatal RDS | NA | NA | alveolar wall thickening with interstitial fibrosis and chronic inflammation; alveoli were filled with macrophages and PAS-positive material | NA | death due to ARDS due to large cell lung carcinoma with widespread metastases (massive involvement of myocardium). |
| Nattes_2017_P10  Borie_2021_P1 | c.267dup | 13 | 40y | ILD , fibrosis | NA | PFT/FEV1 44  PFT/FVC48  PFT/TLC 54  PFT/DLCO 28 | invasive mucinous adenocarcinoma | Systemic steroids, azithromycin, pirfenidone; nintedanib, received chemotherapy with carboplatin and pemetrexed | Listed for DLTX |

**Asthma / recurrent bronchial obstruction / wheeze**

| **Patient and reference** | **Genotype** | **Number** | **Age at pulmonary manifestations (years)** | **First pulmonary symptoms** | **X-ray or CT diagnosis** | **Other diagnosis procedure** | **Lung biopsy** | **Treatment** | **Follow-up** |
| --- | --- | --- | --- | --- | --- | --- | --- | --- | --- |
| Gras_2012_P1 | c.257dupA | 26 | NA | asthma | NA | NA | NA | NA | NA |
| Gras_2012_P2 | c.257dupA | 27 | NA | asthma | NA | NA | NA | NA | NA |
| Gras_2012_P11 | c737+1_373+4del | 29 | NA | asthma | NA | NA | NA | NA | NA |
| Gras_2012_P15 | c581T>G | 30 | NA | asthma | NA | NA | NA | NA | NA |
| Gras_2012_P16 | c581T>G | 31 | NA | asthma | NA | NA | NA | NA | NA |
| Gras_2012_P17 | c581T>G | 32 | NA | asthma | NA | NA | NA | NA | NA |
| Gras_2012_P18 | c581T>G | 33 | NA | asthma | NA | NA | NA | NA | NA |
| Gras_2012_P19 | c581T>G | 34 | NA | asthma | NA | NA | NA | NA | NA |
| Gras_2012_P20 | c581T>G | 35 | NA | asthma | NA | NA | NA | NA | NA |
| Gras_2012_P21 | c.399delC | 36 | NA | asthma | NA | NA | NA | NA | NA |
| Gras_2012_P22 | c.526C>G | 37 | NA | asthma | NA | NA | NA | NA | NA |
| Koht_2016_II:4 | c.671T>G | 88 | 36 | asthma | NA | NA | NA | NA | NA |
| Koht_2016_II:7 | c.671T>G | 89 | NA | asthma | NA | NA | NA | NA | NA |
| Koht_2016_III:6 | c.671T>G | 90 | NA | asthma | NA | NA | NA | NA | NA |
| Koht_2016_IV:1 | c.671T>G | 92 | NA | asthma | NA | NA | NA | NA | NA |
| Koht_2016_IV:2 | c.671T>G | 93 | NA | asthma | NA | NA | NA | NA | NA |
| Koht_2016_IV:3 | c.671T>G | 94 | NA | asthma | NA | NA | NA | NA | NA |
| Maric_2020_P1 | c.254dupG | 95 | neonatal | neonatal RDS | decreased transparency, resembling ground glass with bilateral pulmonary infiltrates | NA | NA | Neonatal invasive ventilation for 12 days, O_2_ supplementation until the age of 11 months, systemic steroid | recurrent infection with wheezing |
| Parnes_2019_P1 | c.754_755insT | 96 | neonatal | neonatal RDS | NA | NA | NA | Neonatal invasive ventilation | recurrent infection with wheezing |
| Parnes_2019_P4 | c.344delG | 99 | neonatal | neonatal RDS | NA | NA | NA | Neonatal invasive ventilation | recurrent infection with wheezing |
| Thorwarth_2014_P7 | c.608C>G | 117 | NA | NA | NA | NA | NA | NA | NA |
| Thorwarth_2014_P9 | c.608C>G | 119 | NA | NA | NA | NA | NA | NA | NA |
| Thorwarth_2014_P13 | c.506C>A | 123 | NA | NA | NA | NA | NA | NA | NA |
| Thorwarth_2014_P22 | deletion | 132 | <1y | NA | NA | NA | NA | NA | NA |
| Glik_2008_PIII:2 | c.650C>A | 144 | NA | Asthma | NA | NA | NA | NA | NA |
| Devos_2006_PII3 | deletion | 147 | 2 | Asthma | NA | NA | NA | Systemic steroids | NA |
| Devos_2006_PIII6 | deletion | 148 | <1y | Neonatal RDS | NA | NA | NA | O_2_ supplementation (10d), invasive ventilation | NA |

D: days, ECMO: extracorporeal membrane oxygenation, GGO: ground glass opacity, HCQ: hydroxychloroquine, HFO: high-frequency oscillation, iNO: inhaled Nitric Oxide, mo: months, NIV: non-invasive ventilation, y: years.

**Pulmonary hypertension**

| **Patient and reference** | **Genotype** | **Number** | **Age at pulmonary manifestations (years)** | **First pulmonary symptoms** | **X-ray or CT diagnosis** | **Other diagnosis procedure** | **Lung biopsy** | **Treatment** | **Follow-up** |
| --- | --- | --- | --- | --- | --- | --- | --- | --- | --- |
| Hamvas_2012_PC | Large deletion | 43 | neonatal | neonatal RDS | NA | NA | NA | O_2_ supplementation (5y), invasive ventilation, ECMO | no oxygen, recurrent upper airway infections with bronchial obstruction |
| Hamvas_2012_PD | Large deletion | 44 | neonatal | neonatal RDS | NA | NA | NA | O_2_ supplementation (1y) | asymptomatic |
| Hamvas_2012_PI | c.583C>T | 49 | neonatal | neonatal RDS (refractory) | NA | NA | growth abnormality with alveolar enlargement and simplification | O_2_ supplementation (8mo), DLTX | death due to PH |
| Hamvas_2012_PL1 | c.592T>C | 52 | 7y | hypoxemia, ILD | NA | NA | NA |  | PH, obstructive lung disease |
| Hamvas_2012_PQ | c.1044_1045insC | 59 | neonatal | neonatal RDS | CT: diffuse ground glass opacification and patchy consolidation | NA | NA | O_2_ supplementation (4y) | asymptomatic |
| Hamvas_2012_PR | c.1092_1108del17 | 60 | neonatal | neonatal RDS | NA | BAL fluid: abnormally low amount of surfactant protein C (SP-C) in relation to SP-B, and low levels of surfactant phospholipids | NA |  | death |
| Kleinlein_2010 | c.278_306del29 | 62 | neonatal | neonatal RDS | X-ray: diffuse GGO increasing from d3 to d13 | NA |  | O_2_ supplementation (10mo), invasive ventilation, HFO (4mo), iNO, short-term antibiotic treatment, systemic steroids, HCQ | death due to respiratory failure and right heart failure |
| Lynn_2020_P1 | del14q13.1–14q21.1 | 69 | neonatal | neonatal RDS | X-ray: multifocal pulmonary opacities with coarse interstitial markings and right upper lobe atelectasis. CT: multifocal pulmonary opacities with coarse interstitial markings and right upper lobe atelectasis | NA | NA | O_2_ supplementation (14mo), NIV, invasive ventilation, short-term antibiotic treatment, Corosurf | hypoxemia, start BIPAP due to hypercapnia |
| Prasad_2019_P1 | del14q13.2-14q21.1 | 76 | neonatal | neonatal RDS | X-ray: Diffuse granular shadowing in both lungs | NA | NA | O_2_ supplementation (25d), NIV (8d), invasive ventilation (7d), iNO (7d), short-term antibiotic treatment | recurrent infections |
| Villamil-Osorio_2021_P1 | del14q12q21.1 | 78 | neonatal | neonatal RDS | CT: diffuse patchy GGO with focal consolidations | BAL fluid: positive for E. coli | non-specific interstitial pneumonia | Invasive ventilation, Corosurf | NA |
| Maric_2020_P1 | c.254dupG | 95 | neonatal | neonatal RDS | X-ray: decreased transparency, resembling ground glass with bilateral pulmonary infiltrates | NA | NA | O_2_ supplementation (11mo), invasive ventilation (12d), systemic steroids | recurrent infection with wheezing |
| Parnes_2019_P2 | c.390C>G | 97 | neonatal | neonatal RDS | NA | NA | NA | Invasive ventilation | asymptomatic |
| Parnes_2019_P3 | c.390C>G | 98 | neonatal | neonatal RDS | NA | NA | NA | Invasive ventilation | asymptomatic |
| Parnes_2019_P4 | c.344delG | 99 | neonatal | neonatal RDS | NA | NA | NA | Invasive ventilation | recurrent wheezing |
| Maquet_2009_P1 | c.619A>C | 103 | neonatal | neonatal RDS | NA | NA | pulmonary tissue with low alveolar counts; simplification of the pulmonary architecture with impaired pulmonary branching, and a morphology | O_2_ supplementation (40d), invasive ventilation, Corosurf | death due to respiratory failure |

D: days, ECMO: extracorporeal membrane oxygenation, GGO: ground glass opacity, HCQ: hydroxychloroquine, HFO: high-frequency oscillation, iNO: inhaled Nitric Oxide, mo: months, NIV: non-invasive ventilation, y: years.

**Chronic respiratory insufficiency**

| **Patient and reference** | **Genotype** | **Number** | **Age at pulmonary manifestations (years)** | **First pulmonary symptoms** | **X-ray or CT diagnosis** | **Other diagnosis procedure** | **Lung biopsy** | **Treatment** | **Follow-up** |
| --- | --- | --- | --- | --- | --- | --- | --- | --- | --- |
| Nattes_2017_P4 | c.583C>T | 7 | Neonatal | neonatal RDS | NA | NA | y; septal thickening, intra-alveolar macrophage accumulation, hyperplasia of alveolar type II cells, and presence of amorphous material stained with periodic acid-Schiff | O_2_ supplementation (18mo), invasive ventilation, oral steroids followed by 16 pulses | death |
| Nattes_2017_P5 | c.876_877del | 8 | Neonatal | neonatal RDS | CT: bilateral alveolar consolidations and ground-glass opacities | BGA: hypoxemia | No | O_2_ supplementation (10y), invasive ventilation, oral steroids followed by 45 pulses, azithromycin, HCQ | hypoxemia |
| Nattes_2017_P6 | c.463+2T>C | 9 | Neonatal | neonatal RDS | NA | BGA: hypoxemia | No | O_2_ supplementation (2y) , NIV, oral and pulse steroids, azithromycin, HCQ | dyspnoea at rest |
| Nattes_2017_P7 | c.344dup | 10 | Neonatal | neonatal RDS | NA | NA | No | O_2_ supplementation (27mo), invasive ventilation | hypoxemia |
| Nattes_2017_P8 | c.175_176del | 11 | 7mo | ILD | CT: heterogenous ground glass opacities | NA | No | O_2_ supplementation (2,5y) | hypoxemia |
| Nattes_2017_P9 | c.572G>T | 12 | 4mo | ILD | CT: paraseptal emphysema, widespread cystic airspaces, diffuse ground glass opacities | BGA: hypoxemia  PFT/FEV1 45 PFT/FVC 45 | y; normal findings | O_2_ supplementation (10y) | dyspnoea at rest |
| Nattes_2017_P13 | c.728G>A | 16 | Neonatal | neonatal RDS | NA | NA | No | O_2_ supplementation (4 mo) | hypoxemia |
| Nattes_2017_P15 | Del14q13.3q21.1 | 18 | 7y | ILD | NA | BGA: hypoxemia | y; septal thickening, intra-alveolar macrophage accumulation, hyperplasia of alveolar type II cells, and presence of amorphous material stained with periodic acid-Schiff | O_2_ supplementation (4mo) | hypoxemia, respiratory exacerbation |
| Galambos_2010_P1 | del14q12q21.3 | 24 | Neonatal | neonatal RDS | X-ray: diffuse granular opacification, positive air bronchogram, CT: bilateral cysts predominantly posteriorly and symmetrically distributed | NA | y; simplified enlarged alveoli with short alveolar crest | O_2_ supplementation (8mo) | death |
| Guillot_2010_P1 | c.493C>T | 39 | 1mo | neonatal RDS | CT: bilateral GGO, focal consolidations predominantly right lung | BAL: neutrophilic inflammation, decreased macrophages, BGA: hypoxemia | normal bronchioles, septal and luminal alveolitis with endoluminal macrophage accumulation and alveolar type 2 hyperplasia, accumulation of amorphous material PAS+ | O_2_ supplementation  (18mo) | death due to respiratory failure |
| Guillot_2010_P2 | c.786_787del2 | 40 | 1mo | neonatal RDS | CT: bilateral GGO, focal consolidations predominantly lung bases | BAL: neutrophilic inflammation, decreased macrophages, decreased SP-B and SP-C, BGA: hypoxemia | No | O_2_ supplementation  (24mo) | hypoxia |
| Hamvas_2012_PA | Large deletion | 41 | 2 | hypoxemia, recurrent infections | CT: ground glass and irregular opacities, pleural based cystic, peribronchial thickening, and bronchiectasis | NA | No | O_2_ supplementation | hypoxia, recurrent pneumothoraxes |
| Hamvas_2012_PC | Large deletion | 43 | Neonatal | neonatal RDS | NA | NA | No | O_2_ supplementation  (5y) | no oxygen, recurrent upper airway infections with bronchial obstruction |
| Hamvas_2012_PD | Large deletion | 44 | Neonatal | neonatal RDS | NA | NA | No | O_2_ supplementation  (1y) | asymptomatic |
| Hamvas_2012_PE | deletion exon 1 and 2 | 45 | Neonatal | neonatal RDS (severe) | NA | NA | hyperplastic pneumocytes with normal lamellar bodies, cytoplasmic heterogeneous dense structures some containing lamellar body like membranes and small vacuoles, and composite lamellar body and dense structures | O_2_ supplementation  (2,8y) | hypoxia |
| Hamvas_2012_PF | c.384_391del8 | 46 | 12mo | severe infection (RSV, influenza A) | CT: ground glass and irregular opacities, pleural based cystic, peribronchial thickening, and bronchiectasis | NA | normal or only minimally altered architecture | O_2_ supplementation  (3y) | asymptomatic |
| Hamvas_2012_PG | c.432C>A | 47 | 4mo | hypoxemia, recurrent infections | NA | NA | severe airway lobular injury and repair (RSV infection) |  | death due to RSV infection |
| Hamvas_2012_PH | c.552_556del | 48 | Neonatal | neonatal RDS | NA | NA | minimal changes without alveolar enlargement | O_2_ supplementation  (19mo) | hypoxia |
| Hamvas_2012_PI | c.583C>T | 49 | Neonatal | neonatal RDS (refractory) | NA | NA | growth abnormality with alveolar enlargement and simplification | O_2_ supplementation  (8mo) | death due to PH |
| Hamvas_2012_PJ | c.590T>C | 50 | Neonatal | neonatal RDS | NA | NA | representative of a surfactant dysfunction mutation with hyperplastic Type 2 pneumocytes, alveolar macrophage accumulation, and interstitial thickening | O_2_ supplementation  (10mo) | good |
| Hamvas_2012_PK | c.592T>C | 51 | 12mo | hypoxemia, ILD | NA | NA | mild to moderate alveolar growth disorder, marked chronic lobular remodelling, focal foamy macrophages, focal mild alveolar epithelial hyperplasia | NA | death due to rejection |
| Hamvas_2012_PL1 | c.592T>C | 52 | 7y | hypoxemia, ILD | NA | NA | No | O_2_ supplementation | PH, obstructive lung disease |
| Hamvas_2012_PM | c.594c>G | 55 | Neonatal | neonatal RDS | NA | NA | No | O_2_ supplementation  (16y) | hypoxia |
| Hamvas_2012_PN | c.804_812dupCGGCGGGGG | 56 | Neonatal | neonatal RDS (refractory) | NA | NA | growth abnormality with alveolar enlargement and simplification | O_2_ supplementation | asymptomatic |
| Hamvas_2012_PO | c.804_812dupCGGCGGGGG | 57 | Neonatal | neonatal RDS | NA | NA | moderate alveolar growth disorder | O_2_ supplementation  (24mo) | asymptomatic |
| Hamvas_2012_PQ | c.1044_1045insC | 59 | Neonatal | neonatal RDS | CT: diffuse ground glass opacification and patchy consolidation | NA | No | O_2_ supplementation  (4y) | asymptomatic |
| Hamvas_2012_PR | c.1092_1108del17 | 60 | Neonatal | neonatal RDS | NA | NA | NA | O_2_ supplementation | death |
| Hamvas_2012_PS | c.1157_63dupACTACGG | 61 | Neonatal | neonatal RDS | CT: diffuse ground glass opacification and patchy consolidation | NA | severe chronic lobular remodelling with diffuse foamy macrophages, diffuse alveolar epithelial hyperplasia | O_2_ supplementation | asymptomatic |
| Kleinlein_2010 | c.278_306del29 | 62 | Neonatal | neonatal RDS | X-ray: diffuse GGO increasing from d3 to d13 | BAL: abnormally low amount of surfactant protein C (SP-C) in relation to SP-B, and low levels of surfactant phospholipids | No | O_2_ supplementation  (10m) | death due to respiratory failure and right heart failure |
| LeMoine_2019_P3 | nonsense | 65 | Neonatal | neonatal RDS | X-ray: diffuse hazy opacities, CT: patchy GGO (RUL, RLL), focal consolidations (RUL, RLL) | NA | bronchus-associated lymphoid tissue, pulmonary hypertensive arteriopathy, alveolar growth abnormality, increased alveolar macrophages, interstitial lymphocytic infiltrate | O_2_ supplementation | n/k |
| Lynn_2020_P1 | del14q13.1–14q21.1 | 69 | Neonatal | neonatal RDS | X-ray: multifocal pulmonary opacities with coarse interstitial markings and right upper lobe atelectasis, CT: multiple focal consolidations, diffuse patchy GGO | NA | No | O_2_ supplementation  (14mo) , NIV | hypoxemia, start BIPAP due to hypercapnia |
| Mirza_2022_P1 | c.190C | 70 | Neonatal | tachypnoea | X-ray: parahilar infiltrates, CT: diffuse patchy GGO | NA | No | O_2_ supplementation  (3mo) | asymptomatic |
| Nevel_2016_S1 (Young_2013_P1) | c.572G.T | 71 | 4mo | tachypnoea, hypoxia | CT: diffuse patchy GGO, mosaicism | PFT/FEV1 51  PFT/FVC48  PFT/TLC 89  PFT/DLCO 83  6MWT desaturation | near normal architecture, mild peribronchial lymphocytic aggregates, increased neuroendocrine cells upon bombesin stain | O_2_ supplementation  (17y) | exercise intolerance, crackles |
| Nevel_2016_S3 | c.572G.T | 73 | NA | tachypnoea, hypoxia | CT: small subpleural calcified nodules with pleural thickening, subtle mosaicism | PFT/FEV1 73  PFT/FVC 74  PFT/TLC 110  PFT/DLCO 97 | NA | O_2_ supplementation  (4y) | asymptomatic since age of 20y |
| Gu_2020_P1 | c.1124_1125insAGGTGGATAC | 85 | Neonatal | neonatal RDS | X-ray: pneumothorax, infiltrate on the right side initially, later on diffuse opacities, CT: GGO, focal consolidations | NA | No | O_2_ supplementation  (3mo) , NIV | death due to respiratory insufficiency |
| Maric_2020_P1 | c.254dupG | 95 | Neonatal | neonatal RDS | X-ray: decreased transparency, resembling ground glass with bilateral pulmonary infiltrates | NA | No | O_2_ supplementation  (11mo) | recurrent infection with wheezing |
| Safi_2017_PIII-1 | c.464-9C>A | 101 | Neonatal (4m) | tachypnoea, bilateral crackles, hypoxia | CT: dependent atelectasis, otherwise normal lung | NA | No | O_2_ supplementation  (3y) | hypoxia |
| Jovien_2016_child | c.373+2T>C | 108 | Neonatal | severe neonatal RDS | CT (16 mo): diffuse ground-glass attenuations | NA | NA | O_2_ supplementation  (2y) | Oxygenotherapy was discontinued |
| Jovien_2016_mother | c.373+2T>C | 109 | Infancy | chronic cough during infancy | CT (27y): fibrosing interstitial pattern with bilateral septal thickening and mild groundglass attenuations. | NA | NA |  | After percutaneous foramen oval closure, her respiratory condition improved partially |

GGO: ground glass opacity, mo: months, , NIV: non-invasive ventilation, y: years

**Interstitial lung disease without neonatal RDS (30)**

| **Patient and reference** | **Genotype** | **Number** | **Age at pulmonary manifestations (years)** | **First pulmonary symptoms** | **X-ray or CT diagnosis** | **Other diagnosis procedure** | **Lung biopsy** | **Treatment** | **Follow-up** |
| --- | --- | --- | --- | --- | --- | --- | --- | --- | --- |
| Nattes_2017_P8 | c.175_176del | 11 | 7mo | ILD | CT: heterogenous ground glass opacities | NA | No | O_2_ supplementation  (2,5y), systemic steroids (27 pulses), azithromycin | hypoxemia |
| Nattes_2017_P9 | c.572G>T | 12 | 4mo | ILD | CT: paraseptal emphysema, widespread cystic airspaces, diffuse ground glass opacities | BGA: hypoxemia  PFT/FEV1 45 PFT/FVC 45 | Normal findings | O_2_ supplementation  (10y) , systemic steroids (72 pulses), azithromycin | dyspnoea at rest |
| Nattes_2017_P10 Borie_2021_P1 | c.267dup | 13 | 40y | ILD , fibrosis | CT: paraseptal emphysema, septal thickening and fibrosis | BGA: normal  PFT/FEV1 44  PFT/FVC 48  PFT/TLC 54  PFT/DLCO 28  6MWT | No | Oral steroids (6w), azithromycin, pirfenidone/ nintedanib | end stage respiratory failure; lung cancer; death due to exacerbation |
| Nattes_2017_P11 | c.463+2T>C | 14 | 25y | ILD with resp. Insufficiency | NA | BGA: hypoxemia  PFT/FEV1 63  PFT/FVC 72  PFT/TLC 95  PFT/DLCO 71  6MWT | No | Oral steroids (2mo), azithromycin, HCQ | hypoxemia, dyspnoea |
| Nattes_2017_P12 | c.175_176del | 15 | 30y | ILD | NA | BGA: normal  PFT/FEV1 80  PFT/FVC88  PFT/TLC 93  PFT/DLCO 66  6MWT | No | NA | paucisymptomatic |
| Nattes_2017_P15 | Del14q13.3q21.1 | 18 | 7y | ILD | NA | BGA: hypoxemia  6MWT | Septal thickening, intra-alveolar macrophage accumulation, hyperplasia of alveolar type II cells, and presence of amorphous material stained with periodic acid-Schiff | O_2_ supplementation  (4mo) , oral and systemic steroids, azithromycin | hypoxemia, respiratory exacerbation |
| Guillot_2010_P1 | c.493C>T | 39 | 1mo | neonatal RDS | CT: bilateral GGO, focal consolidations predominantly right lung | **NA** | normal bronchioles, septal and luminal alveolitis with endoluminal macrophage accumulation and alveolar type 2 hyperplasia, accumulation of amorphous material PAS+ | O_2_ supplementation  (18mo), invasive ventilation (10d), Curosurf (2 doses), oral steroids | death due to respiratory failure |
| Guillot_2010_P2 | c.786_787del2 | 40 | 1mo | neonatal RDS | CT: bilateral GGO, focal consolidations predominantly lung bases | BGA: hypoxemia | No | O_2_ supplementation  (24mo), invasive ventilation (18d), Curosurf (2 doses), oral and systemic steroids (pulses), HCQ | hypoxia |
| Hamvas_2012_PA | Large deletion | 41 | 2 | hypoxemia, recurrent infections | CT: ground glass and irregular opacities, pleural based cystic, peri bronchial thickening, and bronchiectasis | NA |  | O_2_ supplementation | hypoxia, recurrent pneumothoraxes |
| Hamvas_2012_PF | c.384_391del8 | 46 | 12mo | severe infection (RSV, influenza A) | CT: ground glass and irregular opacities, pleural based cystic, peri bronchial thickening, and bronchiectasis | NA | normal or only minimally altered architecture | O_2_ supplementation  (3y) | asymptomatic |
| Hamvas_2012_PG | c.432C>A | 47 | 4mo | hypoxemia, recurrent infections | NA | NA | severe airway lobular injury and repair (RSV infection) | O_2_ supplementation | death due to RSV infection |
| Hamvas_2012_PK | c.592T>C | 51 | 12mo | hypoxemia, ILD | NA | NA | mild to moderate alveolar growth disorder, marked chronic lobular remodelling, focal foamy macrophages, focal mild alveolar epithelial hyperplasia | DLTX (13y) | death due to rejection |
| Hamvas_2012_PL1 | c.592T>C | 52 | 7y | hypoxemia, ILD | NA | NA | No | NA | PH, obstructive lung disease |
| Hamvas_2012_PL2 | c.592T>C | 53 | 26y | pulmonary fibrosis | NA | NA | No | NA | death due to other causes (MVA) |
| LeMoine_2019_P4 | Nonsense | 66 | neonatal | hypoxia, cyanosis | CT: architectural distortion, diffuse patchy GGO, mosaic air-trapping | NA | bronchus-associated lymphoid tissue, pulmonary hypertensive arteriopathy, alveolar growth abnormality, increased alveolar macrophages | O_2_ supplementation, invasive ventilation, short-term antibiotic treatment, systemic steroids | n/k |
| LeMoine_2019_P6 | deletion | 68 | 27mo | wheezing | X-ray: persistent opacity, CT: diffuse GGO, focal consolidations, architectural distortion, hilar adenopathy | NA | No | O_2_ supplementation, short-term antibiotic treatment, ICS, azithromycin | n/k |
| Mirza_2022_P1 | c.190C | 70 | neonatal | tachypnoea | X-ray: parahilar infiltrates, CT: diffuse patchy GGO | NA | No | O_2_ supplementation (3mo), short-term antibiotic treatment, systemic steroids, azithromycin | asymptomatic |
| Nevel_2016_S1 (Young_2013_P1) | c.572G.T | 71 | 4mo | tachypnoea, hypoxia | CT: diffuse patchy GGO, mosaicism | PFT/FEV1 51  PFT/FVC48  PFT/TLC 89  PFT/DLCO 83  6MWT desaturation | near normal architecture, mild peri bronchial lymphocytic aggregates, increased neuroendocrine cells upon bombesin stain | O_2_ supplementation (17y), systemic steroids | exercise intolerance, crackles |
| Nevel_2016_S2 | c.572G.T | 72 | NA | tachypnoea, hypoxia | CT: subtle mosaicism lower lobes, lingula and middle lobe | PFT/FEV1 98  PFT/FVC 99  PFT/TLC 99  PFT/DLCO 102 | No | Short-term antibiotic treatment, ICS | exercise intolerance |
| Nevel_2016_S3 | c.572G.T | 73 | NA | tachypnoea, hypoxia | CT: small subpleural calcified nodules with pleural thickening, subtle mosaicism | PFT/FEV1 73  PFT/FVC 74  PFT/TLC 110  PFT/DLCO 97 | No | O_2_ supplementation (4d), short-term antibiotics | asymptomatic since age of 20y |
| Nevel_2016_S4 | c.572G.T | 74 | NA | tachypnoea, hypoxia | CT: subpleural nodules and calcifications, pleural abnormalities with thickening along major fissure, nodularity of the pleura, subtle mosaicism | PFT/FEV1 73  PFT/FVC 86  PFT/TLC 86  PFT/DLCO 91 | No | Short-term antibiotics | minimal exercise intolerance |
| Nevel_2016_S5 | c.572G.T | 75 | 2mo | tachypnoea, hypoxia | CT: GGO, NEHI pattern usual | NA | No | NA | NA |
| Safi_2017_PIII-1 | c.464-9C>A | 96 | 4mo | tachypnoea, bilateral crackles, hypoxia | CT: dependent atelectasis, otherwise normal lung | BAL: diffuse mixed ground-glass and ill-defined nodular opacities at 20m | No | O_2_ supplementation (3y), invasive ventilation, ECMO (14d), short-term antibiotics, systemic steroids, azithromycin, HCQ | hypoxia |
| Jovien_2016_mother | c.373+2T>C | 104 | infancy | chronic cough during infancy | CT (27y): fibrosing interstitial pattern with bilateral septal thickening and mild ground glass attenuations. | NA | NA | Oral corticosteroids, azithromycin, HCQ | After percutaneous foramen oval closure, her respiratory condition improved partially |
| Sutton_2022_213 | c.67G>C | 106 | 46y | NA | CT: usual interstitial pneumonia | NA | Usual interstitial pneumonia | NA | pulmonary fibrosis |
| Sutton_2022_104 | c.396C>A | 107 | 64y | NA | CT: usual interstitial pneumonia and fibrosis | NA | Usual interstitial pneumonia and emphysema | NA | pulmonary fibrosis |
| Sutton_2022_228 | c.532G>A | 108 | 70y | NA | CT: usual interstitial pneumonia | NA | NA | NA | pulmonary fibrosis |
| Sutton_2022_523 | c.631A>G | 109 | 43y | NA | NA | NA | Usual interstitial pneumonia | NA | pulmonary fibrosis |
| Sutton_2022_057 | c.781C>A | 110 | 73y | NA | NA | NA | NA | NA | pulmonary fibrosis |
| Devos_2006_PII3 | deletion | 147 | 2y | asthma | NA | NA | No | Systemic steroids | hypoxemia |

d: days, ECMO: extracorporeal membrane oxygenation, GGO: ground glass opacity, HCQ: hydroxychloroquine, HFO: high-frequency oscillation, iNO: inhaled Nitric Oxide, mo: months, NIV: non-invasive ventilation, y: years.

**Recurrent infections (62)**

| **Patient and reference** | **Genotype** | **Number** | **Age at pulmonary manifestations (years)** | **First pulmonary symptoms** | **X-ray or CT diagnosis** | **Other diagnosis procedure** | **Lung biopsy** | **Treatment** | **Follow-up** |
| --- | --- | --- | --- | --- | --- | --- | --- | --- | --- |
| Carre_2009_P1 | del14q13 | 1 | neonatal | neonatal RDS | NA | NA | No | NA | NA |
| Carre_2009_P2 | intronic splice site mutation at 376-2A.G | 2 | neonatal | neonatal RDS | NA | NA | No | NA | NA |
| Nattes_2017_P15 | Del14q13.3q21.1 | 18 | 7y | ILD | NA | BGA: hypoxemia  6MWT | y; septal thickening, intra-alveolar macrophage accumulation, hyperplasia of alveolar type II cells, and presence of amorphous material stained with periodic acid-Schiff | O_2_ supplementation  (4mo) , oral and systemic steroids, azithromycin | Hypoxemia, respiratory exacerbation |
| Galambos_2010_P1 | del14q12q21.3 | 24 | neonatal | neonatal RDS | X-ray: diffuse granular opacification, positive air bronchogram, CT: bilateral cysts predominantly posteriorly and symmetrically distributed | NA | y; simplified enlarged alveoli with short alveolar crest | O_2_ supplementation  (8m), invasive ventilation (22d), HFO (14d), iNO (7d), Curosurf | Death |
| Hamvas_2012_PA | Large deletion | 41 | 2 | hypoxemia, recurrent infections | CT: ground glass and irregular opacities, pleural based cystic, peri bronchial thickening, and bronchiectasis | NA | NA | O_2_ supplementation | Hypoxia, recurrent pneumothoraxes |
| Hamvas_2012_PB | Large deletion | 42 | neonatal | neonatal RDS | NA | NA | NA | NA | Recurrent infections |
| Hamvas_2012_PC | Large deletion | 43 | neonatal | neonatal RDS | NA | NA | NA | NA | No oxygen, recurrent upper airway infections with bronchial obstruction |
| Hamvas_2012_PG | c.432C>A | 47 | 4 months | hypoxemia, recurrent infections | NA | NA | Severe airway lobular injury and repair (RSV infection) | O_2_ supplementation | Death due to RSV infection |
| Hamvas_2012_PH | c.552_556del | 48 | neonatal | neonatal RDS | NA | NA | Minimal changes without alveolar enlargement | O_2_ supplementation  (19mo), invasive ventilation, ECMO | Hypoxia |
| Hamvas_2012_PI | c.583C>T | 49 | neonatal | neonatal RDS (refractory) | NA | NA | growth abnormality with alveolar enlargement and simplification | O_2_ supplementation  (8mo), DLTX (8mo) | Death due to PH |
| Hamvas_2012_PJ | c.590T>C | 50 | neonatal | neonatal RDS | NA | NA | Representative of a surfactant dysfunction mutation with hyperplastic Type 2 pneumocytes, alveolar macrophage accumulation, and interstitial thickening | O_2_ supplementation  (10mo), DLTX (10mo) | Good |
| Hamvas_2012_PL3 | c.592T>C | 54 | neonatal | neonatal RDS | NA | NA | NA | O_2_ supplementation | Died of respiratory failure |
| Hamvas_2012_PM | c.594c>G | 55 | neonatal | neonatal RDS | NA | NA | NA | O_2_ supplementation  (16y) | Hypoxia |
| Hamvas_2012_PO | c.804_812dupCGGCGGGGG | 57 | neonatal | neonatal RDS | NA | NA | Moderate alveolar growth disorder | O_2_ supplementation  (24mo) | Asymptomatic |
| Hamvas_2012_PS | c.1157_63dupACTACGG | 61 | neonatal | neonatal RDS | CT: diffuse ground glass opacification and patchy consolidation | NA | Severe chronic lobular remodelling with diffuse foamy macrophages, diffuse alveolar epithelial hyperplasia | O_2_ supplementation | Asymptomatic |
| Lynn_2020_P1 | del14q13.1–14q21.1 | 69 | neonatal | neonatal RDS | X-ray: multifocal pulmonary opacities with coarse interstitial markings and right upper lobe atelectasis, CT: multiple focal consolidations, diffuse patchy GGO | **NA** | No | O_2_ supplementation (14mo), NIV, invasive ventilation, short-term antibiotic treatment | Hypoxemia, start BIPAP due to hypercapnia |
| Nevel_2016_S1 (Young_2013_P1) | c.572G>T | 71 | 4mo | tachypnoea, hypoxia | CT: diffuse patchy GGO, mosaicism | PFT/FEV1 51  PFT/FVC48  PFT/TLC 89  PFT/DLCO 83  6MWT desaturation | Near normal architecture, mild peri bronchial lymphocytic aggregates, increased neuroendocrine cells upon bombesin stain | O_2_ supplementation  (17y) | Exercise intolerance, crackles |
| Nevel_2016_S2 | c.572G>T | 72 | NA | tachypnoea, hypoxia | CT: subtle mosaicism lower lobes, lingula and middle lobe | PFT/FEV1 98  PFT/FVC 99  PFT/TLC 99  PFT/DLCO 102 | NA | Short-term antibiotic treatment, ICS | Exercise intolerance |
| Nevel_2016_S3 | c.572G>T | 73 | NA | tachypnoea, hypoxia | CT: small subpleural calcified nodules with pleural thickening, subtle mosaicism | PFT/FEV1 73  PFT/FVC 74  PFT/TLC 110  PFT/DLCO 97 | NA | O_2_ supplementation  (4y) | Asymptomatic since age of 20y |
| Nevel_2016_S4 | c.572G>T | 74 | NA | tachypnoea, hypoxia | CT: subpleural nodules and calcifications, pleural abnormalities with thickening along major fissure, nodularity of the pleura, subtle mosaicism | PFT/FEV1 73  PFT/FVC 86  PFT/TLC 86  PFT/DLCO 91 | NA | Short-term antibiotics | minimal exercise intolerance |
| Nevel_2016_S5 | c.572G>T | 75 | 2mo | tachypnoea, hypoxia | CT: GGO, NEHI pattern usual | NA | NA | NA |  |
| Prasad_2019_P1 | del14q13.2-14q21.1 | 76 | neonatal | neonatal RDS | X-ray: Diffuse granular shadowing in both lungs consistent with respiratory distress syndrome | NA | NA | O_2_ supplementation  (25d), NIV (8d), invasive ventilation (7d), iNO (7d), short-term antibiotic treatment | recurrent infections |
| Barnett_2012_P1 | 14q13.1–q13.3 | 79 | neonatal | neonatal RDS | NA | NA | NA | O_2_ supplementation  (2d), short-term antibiotic treatment | NA |
| Hu_2019_P1 | 14q13.2q21.1 | 82 | neonatal | neonatal RDS | CT: interstitial and parenchymal lesions, bronchiectasis | NA | NA | O_2_ supplementation, short-term antibiotic treatment | Chronic cough with expectoration for 3 y |
| Koht_2016_III:7 | c.671T>G | 86 | NA | recurrent infections | NA | NA | NA | NA | NA |
| Maric_2020_P1 | c.254dupG | 90 | neonatal | neonatal RDS | X-ray: decreased transparency, resembling ground glass with bilateral pulmonary infiltrates | NA | No | Neonatal invasive ventilation for 12 days, O_2_ supplementation until the age of 11 months, systemic steroid | Recurrent infection with wheezing |
| Parnes_2019_P1 | c.754_755insT | 91 | neonatal | neonatal RDS | NA | NA | No | Neonatal invasive ventilation | Recurrent infections, wheezing |
| Safi_2017_PIII-1 | c.464-9C>A | 96 | neonatal (4m) | tachypnoea, bilateral crackles, hypoxia | CT: dependent atelectasis, otherwise normal lung | NA | No | O_2_ supplementation  (3y) | Hypoxia |
| Safi_2017_PIII-2 | c.464-9C>A | 97 | neonatal (4m) | hypoxia | NA | NA | No | O_2_ supplementation  (few days), short-term antibiotic, systemic steroids | No respiratory symptoms after prednisolone weaning |
| Doyle_2004_I1 | c.740A>G | 102 | NA | life-threating episodes of pneumonia | NA | NA | NA | NA | n/k |
| Thorwarth_2014_P1 | c.866_833delinsCTACA | 111 | 0 | NA | NA | NA | NA | NA | NA |
| Thorwarth_2014_P2 | c.793delA | 112 | NA | NA | NA | NA | NA | NA | NA |
| Thorwarth_2014_P3 | c.712delGG | 113 | NA | NA | NA | NA | NA | NA | NA |
| Thorwarth_2014_P4 | c.712delGG | 114 | 0 | NA | NA | NA | NA | NA | NA |
| Thorwarth_2014_P5 | c.613G>T | 115 | 0 |  | NA | NA | NA | NA | NA |
| Thorwarth_2014_P6 | c.608C>G + c.613G>T | 116 | NA | NA | NA | NA | NA | NA | NA |
| Thorwarth_2014_P8 | c.608C>G | 118 | NA | NA | NA | NA | NA | NA | NA |
| Thorwarth_2014_P10 | c.585nsGG | 120 | NA | NA | NA | NA | NA | NA | NA |
| Thorwarth_2014_P11 | c.522C>G | 121 | 0 | NA | NA | NA | NA | NA | NA |
| Thorwarth_2014_P12 | c.522C>G | 122 | 0 | NA | NA | NA | NA | NA | NA |
| Thorwarth_2014_P13 | c.506C>A | 123 | NA | NA | NA | NA | NA | NA | NA |
| Thorwarth_2014_P14 | c.338G>A | 124 | NA | NA | NA | NA | NA | NA | NA |
| Thorwarth_2014_P15 | c.261C>A | 125 | 0 | NA | NA | NA | NA | NA | NA |
| Thorwarth_2014_P16 | c.261C>A | 126 | 0 | NA | NA | NA | NA | NA | NA |
| Thorwarth_2014_P18 | c.157insC | 128 | 0 | NA | NA | NA | NA | NA | NA |
| Thorwarth_2014_P19 | deletion | 129 | NA | NA | NA | NA | NA | NA | NA |
| Thorwarth_2014_P20 | deletion | 130 | NA | NA | NA | NA | NA | NA | NA |
| Thorwarth_2014_P21 | deletion | 131 | NA | NA | NA | NA | NA | NA | NA |
| Thorwarth_2014_P22 | deletion | 132 | 0 | NA | NA | NA | NA | NA | NA |
| Thorwarth_2014_P23 | deletion | 133 | 0 | NA | NA | NA | NA | NA | NA |
| Asmus_2005_P1 (III:1) | c.523G>T | 134 | neonatal | neonatal RDS | NA | NA | NA | NA | NA |
| Asmus_2005_P2 (III:2) | c.523G>T | 135 | neonatal | insufficient respiration | NA | NA | NA | NA | NA |
| Asmus_2005_P3 (III:4) | c.523G>T | 136 | neonatal | neonatal RDS | NA | NA | NA | NA | NA |
| Willemsen_2005_P1 | c.859-860insC | 139 | neonatal (2w) | neonatal RDS | NA | NA | Alveolar wall thickening with interstitial fibrosis and chronic inflammation; alveoli were filled with macrophages and PAS-positive material | NA | Death due to ARDS due to large cell lung carcinoma with widespread metastases (massive involvement of myocardium) |
| Peall_2013_P2 | c.522delC | 140 | n/k | recurrent infections | NA | NA | NA | NA | NA |
| Peall_2013_P4 | deletion | 142 | n/k | recurrent infections | NA | NA | NA | NA | NA |
| Iwatani_2000_P1 | del14q12-13.3 | 145 | neonatal (4m) | bronchiolitis | NA | NA | NA | O_2_ supplementation, NIV, invasive ventilation, iNO, Curosurf | recurrent infections |
| Iwatani_2000_P2 | del14q12-13.3 | 146 | neonatal | tachydyspnea, recurrent infections | NA | NA | NA | NA | death due to respiratory failure at 3y |
| Devos_2006_PIII6 | deletion | 148 | 0 | neonatal RDS | NA | NA | NA | O_2_ supplementation (10d), invasive ventilation | NA |

D: days, ECMO: extracorporeal membrane oxygenation, GGO: ground glass opacity, HCQ: hydroxychloroquine, HFO: high-frequency oscillation, iNO: inhaled Nitric Oxide, mo: months, NIV: non-invasive ventilation, y: years.

**Neonatal RDS progressing to ILD**

| **Patient and reference** | **Genotype** | **Number** | **Age at pulmonary manifestations (years)** | **First pulmonary symptoms** | **X-ray or CT diagnosis** | **Other diagnosis procedure** | **Lung biopsy** | **Treatment** | **Follow-up** |
| --- | --- | --- | --- | --- | --- | --- | --- | --- | --- |
| Carre_2009_P1 | del14q13 | 1 | neonatal | neonatal RDS | NA | NA | No | O_2_ supplementation  (3mo), invasive ventilation (8d), long-term antibiotics (several months) | NA |
| Carre_2009_P2 | intronic splice site mutation at 376-2A.G | 2 | neonatal | neonatal RDS | NA | NA | No | NA | NA |
| Carre_2009_P3 | intronic splice site mutation at 376-2A.G | 3 | neonatal | neonatal RDS | NA | NA | No | NA | NA |
| Nattes_2017_P4 | c.583C>T | 7 | neonatal | neonatal RDS | NA | BAL(6/16):  neutrophilic inflammation (3)  lymphocytic inflammation (1)  normal cell count (2)  P5 and P6: hypoxemia  P14:  PFT/FEV1: 84,  PFT/FVC: 120, PFT/TLC: 123, and PFT/DLCO: 93 | y; septal thickening, intra-alveolar macrophage accumulation, hyperplasia of alveolar type II cells, and presence of amorphous material stained with periodic acid-Schiff | 7/10 of neonates received 1-2 doses of Curosurf  P4: O_2_ supplementation  (18mo), invasive ventilation, oral and pulse steroids (16)  P5: O_2_ supplementation  (10y), invasive ventilation, oral and pulse steroids (45), azithromycin, HCQ  P6: O_2_ supplementation  (2y), NIV, oral and pulse steroids (17), azithromycin, HCQ  P7: O_2_ supplementation  (27mo), invasive ventilation, pulse steroids (16), azithromycin  P13: O_2_ supplementation  (4mo), invasive ventilation, pulse steroids  P14: invasive ventilation, oral steroids | Death |
| Nattes_2017_P5 | c.876_877del | 8 | neonatal | neonatal RDS | CT: bilateral alveolar consolidations and ground-glass opacities |  | No |  | Hypoxemia |
| Nattes_2017_P6 | c.463+2T>C | 9 | neonatal | neonatal RDS | NA |  | No |  | Dyspnoea at rest |
| Nattes_2017_P7 | c.344dup | 10 | neonatal | neonatal RDS | NA |  | No |  | Hypoxemia |
| Nattes_2017_P13 | c.728G>A | 16 | neonatal | neonatal RDS | NA |  | No |  | Hypoxemia |
| Nattes_2017_P14 | Del14q13q13 | 17 | neonatal | neonatal RDS | NA |  | No |  | Dyspnoea at exertion |
| Galambos_2010_P1 | del14q12q21.3 | 24 | neonatal | neonatal RDS | CT: bilateral cysts predominantly posteriorly and symmetrically distributed | NA | y; simplified enlarged alveoli with short alveolar crest | O_2_ supplementation  (8m), invasive ventilation (22d), HFO (14d), iNO (7d), Curosurf | Death |
| Gillett_2013_P1 | c.621C>G | 25 | neonatal | neonatal RDS | X-ray: bilateral hazy lungs consistent with RDS | NA | diffuse alteration of the architecture by alveolar remodeling and Type II cell hyperplasia. There was alveolar filling by granular proteinaceous material, focally periodic acid-Schiff-positive | O_2_ supplementation, invasive ventilation, HFO, iNO, ECMO, Curosurf | Death |
| Hamvas_2012_PE | deletion exon 1 and 2 | 45 | neonatal | neonatal RDS (severe) | NA | NA | hyperplastic pneumocytes with normal lamellar bodies, cytoplasmic heterogeneous dense structures some containing lamellar body like membranes and small vacuoles, and composite lamellar body and dense structures | O_2_ supplementation (2,8y), systemic steroids, azithromycin, HCQ | Hypoxia |
| Hamvas_2012_PH | c.552_556del | 48 | neonatal | neonatal RDS | NA | NA | minimal changes without alveolar enlargement | O_2_ supplementation (19mo), invasive ventilation, ECMO | Hypoxia |
| Hamvas_2012_PI | c.583C>T | 49 | neonatal | neonatal RDS (refractory) | NA | NA | growth abnormality with alveolar enlargement and simplification | O_2_ supplementation (8mo), invasive ventilation, DLTX (8mo) | Death due to PH |
| Hamvas_2012_PJ | c.590T>C | 50 | neonatal | neonatal RDS | NA | NA | representative of a surfactant dysfunction mutation with hyperplastic Type 2 pneumocytes, alveolar macrophage accumulation, and interstitial thickening | O_2_ supplementation (10mo), DLTX (10mo) | Good |
| Hamvas_2012_PL3 | c.592T>C | 54 | neonatal | neonatal RDS | NA | NA | No | O_2_ supplementation | Died of respiratory failure |
| Hamvas_2012_PM | c.594c>G | 55 | neonatal | neonatal RDS | NA | NA | No | O_2_ supplementation (16y) | Hypoxia |
| Hamvas_2012_PN | c.804_812dupCGGCGGGGG | 56 | neonatal | neonatal RDS (refractory) | NA | NA | growth abnormality with alveolar enlargement and simplification | O_2_ supplementation, DLTX (7mo) | Asymptomatic |
| Hamvas_2012_PQ | c.1044_1045insC | 59 | neonatal | neonatal RDS | CT: diffuse ground glass opacification and patchy consolidation | NA | No | O_2_ supplementation (4Y) | Asymptomatic |
| Hamvas_2012_PR | c.1092_1108del17 | 60 | neonatal | neonatal RDS | NA | NA | No | O_2_ supplementation | Death |
| Hamvas_2012_PS | c.1157_63dupACTACGG | 61 | neonatal | neonatal RDS | CT: diffuse ground glass opacification and patchy consolidation | NA | severe chronic lobular remodeling with diffuse foamy macrophages, diffuse alveolar epithelial hyperplasia | O_2_ supplementation, DLTX (22mo) | Asymptomatic |
| Kleinlein_2010 | c.278_306del29 | 62 | neonatal | neonatal RDS | X-ray: diffuse GGO increasing from d3 to d13 | BAL: abnormally low amount of surfactant protein C (SP-C) in relation to SP-B, and low levels of surfactant phospholipids | No | O_2_ supplementation (10mo), invasive ventilation, HFMO (4mo), iNO, short-term antibiotic treatment, Curosurf, systemic steroids, HCQ | Death due to respiratory failure and right heart failure |
| Lynn_2020_P1 | del14q13.1–14q21.1 | 69 | neonatal | neonatal RDS | X-ray: multifocal pulmonary opacities with coarse interstitial markings and right upper lobe atelectasis, CT: multiple focal consolidations, diffuse patchy GGO | NA | No | O_2_ supplementation (14mo), NIV, invasive ventilation, short-term antibiotic treatment | hypoxemia, start BIPAP due to hypercapnia |
| Gu_2020_P1 | c.1124_1125insAGGTGGATAC | 80 | neonatal | neonatal RDS | X-ray: pneumothorax, infiltrate on the right side initially, later on diffuse opacities, CT: GGO, focal consolidations | NA | No | O_2_ supplementation (3mo), NIV, invasive ventilation, HFO, long-term antibiotic treatment, systemic corticosteroids, azithromycin | death due to respiratory insufficiency |
| Maric_2020_P1 | c.254dupG | 90 | neonatal | neonatal RDS | X-ray: decreased transparency, resembling ground glass with bilateral pulmonary infiltrates | NA | No | O_2_ supplementation (11mo), invasive ventilation (12d), systemic corticosteroids | recurrent infection with wheezing |
| Jovien_2016_child | c.373+2T>C | 103 | neonatal | severe neonatal RDS | CT (16 months): diffuse ground-glass attenuations | NA | No | O_2_ supplementation (2y), oral corticosteroids, azithromycin, HCQ | Oxygen therapy was discontinued |
| Willemsen_2005_P1 | c.859-860insC | 139 | neonatal (2 weeks) | neonatal RDS | NA | NA | No | NA | NA |
| Devos_2006_PIII6 | deletion | 148 | NA | NA | NA | NA | No | O_2_ supplementation, invasive ventilation (10d) | NA |

D: days, ECMO: extracorporeal membrane oxygenation, GGO: ground glass opacity, HCQ: hydroxychloroquine, HFO: high-frequency oscillation, iNO: inhaled Nitric Oxide, mo: months, NIV: non-invasive ventilation, y: years.

**Neonatal RDS not progressing to ILD**

| **Patient and reference** | **Genotype** | **Number** | **Age at pulmonary manifestations (years)** | **First pulmonary symptoms** | **X-ray or CT diagnosis** | **Other diagnosis procedure** | **Lung biopsy** | **Treatment** | **Follow-up** |
| --- | --- | --- | --- | --- | --- | --- | --- | --- | --- |
| Nattes_2017_P1 | c.714G>A | 4 | neonatal | neonatal RDS | NA | 6/16 had BAL  neutrophilic inflammation (3)  lymphocytic inflammation (1)  normal cell count (2) | No | O_2_ supplementation  (25d), invasive ventilation | Healthy |
| Nattes_2017_P2 | c.714G>A | 5 | neonatal | neonatal RDS | NA |  | No | O_2_ supplementation  (1d), invasive ventilation | Asthma |
| Nattes_2017_P3 | c.344dup | 6 | neonatal | neonatal RDS | NA |  | No | O_2_ supplementation  (5d), invasive ventilation | Healthy |
| Nattes_2017_P16 | Del14q12q21 | 19 | neonatal | neonatal RDS | NA |  | No | **NA** | Paucisymptomatic |
| Ferrara_2012_PII-4 | c.617T>A | 20 | neonatal | neonatal RDS | NA | NA | NA | **NA** |  |
| Ferrara_2012_PIII-3 | c.617T>A | 21 | neonatal | neonatal RDS | NA | NA | NA | Invasive ventilation |  |
| Ferrara_2012_PIII-5 | c.617T>A | 22 | neonatal | neonatal RDS | NA | NA | NA | Invasive ventilation |  |
| Ferrara_2012_PIII-7 | c.617T>A | 23 | neonatal | neonatal RDS | NA | NA | NA | Invasive ventilation |  |
| Gras_2012_P23 | c789_787del | 38 | NA | neonatal RDS | NA | NA | NA | **NA** | n/k |
| Guillot_2010_P1 | c.493C>T | 39 | 1 month | neonatal RDS | CT: bilateral GGO, focal consolidations predominantly right lung | **BAL: neutrophilic inflammation, decreased macrophages** | normal bronchioles, septal and luminal alveolitis with endoluminal macrophage accumulation and alveolar type 2 hyperplasia, accumulation of amorphous material PAS+ | O_2_ supplementation  (18mo), invasive ventilation (10 d), Curosurf (2 doses), oral and pulse corticosteroids | Death due to respiratory failure |
| Guillot_2010_P2 | c.786_787del2 | 40 | 1 month | neonatal RDS | CT: bilateral GGO, focal consolidations predominantly lung bases | **BAL: neutrophilic inflammation, decreased macrophages, decreased SP-B and SP-C, hypoxemia** | No | O_2_ supplementation  (24mo), invasive ventilation (18 d), Curosurf (2 doses), oral and pulse corticosteroids, HCQ | Hypoxia |
| Hamvas_2012_PB | Large deletion | 42 | neonatal | neonatal RDS | NA | NA | NA | O_2_ supplementation  (1d) | Recurrent infections |
| Hamvas_2012_PC | Large deletion | 43 | neonatal | neonatal RDS | NA | NA | NA | O_2_ supplementation  (5y), invasive ventilation, ECMO | No oxygen, recurrent upper airway infections with bronchial obstruction |
| Hamvas_2012_PD | Large deletion | 44 | neonatal | neonatal RDS | NA | NA | NA | O_2_ supplementation  (1y) | Asymptomatic |
| Hamvas_2012_PO | c.804_812dupCGGCGGGGG | 57 | neonatal | neonatal RDS | NA | NA | moderate alveolar growth disorder | O_2_ supplementation  (24mo) | Asymptomatic |
| Hamvas_2012_PP | c.818_838del21 and c.1034_1047del14 | 58 | neonatal | neonatal RDS | NA | NA | NA | O_2_ supplementation, invasive ventilation, ECMO | Death |
| LeMoine_2019_P1 | Nonsense | 63 | neonatal | neonatal RDS | CT: diffuse GGO, mosaic air trapping, diffuse interlobular thickening | NA | No | O_2_ supplementation, NIV, ICS, azithromycin | NA |
| LeMoine_2019_P2 | deletion | 64 | neonatal | neonatal RDS | CT: diffuse GGO, focal consolidations, diffuse interlobular septal thickening, architectural distortion, hilar and mediastinal lymphadenopathy | NA | No | O_2_ supplementation, invasive ventilation, ECMO, corticosteroids | NA |
| LeMoine_2019_P3 | Nonsense | 65 | neonatal | neonatal RDS | X-ray: diffuse hazy opacities, CT: patchy GGO (RUL, RLL), focal consolidations (RUL, RLL) | Desaturatio**n** | bronchus-associated lymphoid tissue, pulmonary hypertensive arteriopathy, alveolar growth abnormality, increased alveolar macrophages, interstitial lymphocytic infiltrate | O_2_ supplementation, corticosteroids, azithromycin | NA |
| LeMoine_2019_P5 | Duplication | 67 | neonatal | neonatal RDS | CT: focal consolidations, segmental air-trapping, architectural distortion, bronchiectasis | NA | bronchus-associated lymphoid tissue, pulmonary hypertensive arteriopathy, alveolar growth abnormality, increased alveolar macrophages | O_2_ supplementation, NIV (1d), ICS | NA |
| Prasad_2019_P1 | del14q13.2-14q21.1 | 76 | neonatal | neonatal RDS | X-ray: diffuse granular shadowing in both lungs consistent with respiratory distress syndrome | NA | No | O_2_ supplementation  (25d), NIV (8d), invasive ventilation (7d), iNO (7d), short-term antibiotic treatment | Recurrent infections |
| Salerno_2014_P1 | c.334G>T | 77 | neonatal | neonatal RDS | CT: diffuse patchy GGO, focal consolidation, bronchial wall thickening | BAL: normal | No | O_2_ supplementation  (48d) | NA |
| Villamil-Osorio_2021_P1 | del14q12q21.1 | 78 | neonatal | neonatal RDS | CT: diffuse patchy GGO with focal consolidations | BAL: positive for E. coli | non-specific interstitial pneumonia | O_2_ supplementation, invasive ventilation, Curosurf | NA |
| Barnett_2012_P1 | 14q13.1–q13.3 | 79 | neonatal | neonatal RDS | NA | NA | No | O_2_ supplementation  (2d), short-term antibiotic treatment | NA |
| Hanes_2018_P1 | No specified | 81 | neonatal | neonatal RDS | NA | NA | NA | O_2_ supplementation, invasive ventilation, short-term antibiotic treatment | No respiratory issues |
| Hu_2019_P1 | 14q13.2q21.1 | 82 | neonatal | neonatal RDS | CT: interstitial and parenchymal lesions, bronchiectasis | NA | No | O_2_ supplementation, short-term antibiotic treatment | Chronic cough with expectoration for 3 y |
| Parnes_2019_P1 | c.754_755insT | 91 | neonatal | neonatal RDS | NA | NA | No | O_2_ supplementation, invasive ventilation | Recurrent infections, wheezing |
| Parnes_2019_P2 | c.390C>G | 92 | neonatal | neonatal RDS | NA | NA | No | O_2_ supplementation, invasive ventilation | Asymptomatic |
| Parnes_2019_P3 | c.390C>G | 93 | neonatal | neonatal RDS | NA | NA | No | O_2_ supplementation, invasive ventilation | Asymptomatic |
| Parnes_2019_P4 | c.344delG | 94 | neonatal | neonatal RDS | NA | NA | No | O_2_ supplementation, invasive ventilation | Recurrent wheezing |
| Safi_2017_PII-2 | c.464-9C>A | 95 | neonatal | neonatal RDS | NA | NA | No | O_2_ supplementation, invasive ventilation, ECMO, | NA |
| Maquet_2009_P1 | c.619A>C | 98 | neonatal | neonatal RDS | NA | NA | pulmonary tissue with low alveolar counts; simplification of the pulmonary architecture with impaired pulmonary branching, and a morphology suggestive of surfactant deficiency | O_2_ supplementation (40d), invasive ventilation (40d), Curosurf | Death due to respiratory failure |
| Doyle_2004_III2 | c.740A>G | 99 | neonatal | neonatal RDS | NA | NA | NA | **Invasive ventilation** | NA |
| Doyle_2004_III3 | c.740A>G | 100 | neonatal | neonatal RDS | NA | NA | NA | **Invasive ventilation** | NA |
| Doyle_2004_II2 | c.740A>G | 101 | neonatal | neonatal RDS | NA | NA | NA | O_2_ supplementation (24d) | NA |
| Doyle_2004_I1 | c.740A>G | 102 | NA | life-threating episodes of pneumonia | NA | NA | NA | **NA** | NA |
| Salvado_2013_patient 2 | c.825delC | 105 | neonatal | neonatal RDS | NA | NA | NA | **NA** | NA |
| Thorwarth_2014_P1 | c.866_833delinsCTACA | 111 | NA | NA | NA | NA | NA | **NA** | NA |
| Thorwarth_2014_P4 | c.712delGG | 114 | NA | NA | NA | NA | NA | **NA** | NA |
| Thorwarth_2014_P5 | c.613G>T | 115 | NA | NA | NA | NA | NA | **NA** | NA |
| Thorwarth_2014_P11 | c.522C>G | 121 | NA | NA | NA | NA | NA | **NA** | NA |
| Thorwarth_2014_P12 | c.522C>G | 122 | NA | NA | NA | NA | NA | **NA** | NA |
| Thorwarth_2014_P15 | c.261C>A | 125 | NA | NA | NA | NA | NA | **NA** | NA |
| Thorwarth_2014_P16 | c.261C>A | 126 | NA | NA | NA | NA | NA | **NA** | NA |
| Thorwarth_2014_P17 | c.236C>A | 127 | NA | NA | NA | NA | NA | **NA** | NA |
| Thorwarth_2014_P18 | c.157insC | 128 | NA | NA | NA | NA | NA | **NA** | NA |
| Thorwarth_2014_P22 | deletion | 132 | NA | NA | NA | NA | NA | **NA** | NA |
| Thorwarth_2014_P23 | deletion | 133 | NA | NA | NA | NA | NA | **NA** | NA |
| Asmus_2005_P1 (III:1) | c.523G>T | 134 | Neonatal | neonatal RDS | NA | NA | NA | **NA** | NA |
| Asmus_2005_P2 (III:2) | c.523G>T | 135 | Neonatal | insufficient respiration | NA | NA | NA | **NA** | NA |
| Asmus_2005_P3 (III:4) | c.523G>T | 136 | Neonatal | neonatal RDS | NA | NA | NA | **NA** | NA |
| Pohlenz_2002_P1 | c.254dupG | 137 | Neonatal | neonatal RDS | NA | NA | NA | O_2_ supplementation (14d), invasive ventilation (14d) | NA |
| Ferrara_2008_P1 | c.609C>A | 138 | Neonatal | neonatal RDS | NA | NA | NA | NA | NA |
| Peall_2013_P3 | c.338G>A | 141 | Neonatal | neonatal RDS | NA | NA | NA | NA | NA |
| Peall_2013_P5 | c.782C>T | 143 | Neonatal | neonatal RDS | NA | NA | NA | NA | NA |

D: days, ECMO: extracorporeal membrane oxygenation, GGO: ground glass opacity, HCQ: hydroxychloroquine, HFO: high-frequency oscillation, iNO: inhaled Nitric Oxide, mo: months, NIV: non-invasive ventilation, y: years.
