# Supplementary material for: Respiratory and other organ manifestations in NKX2-1-related disorders: a systematic review
Source: Front Med (Lausanne). 2025 May 6;12:1507513. doi: 10.3389/fmed.2025.1507513 (PMC12090872; doi:10.3389/fmed.2025.1507513)
Supplement: Supplementary file 5 [file Supplementary_file_5.docx]

| **Supplemental Data 5.** Detailed pulmonary function test results | | | | | | | | | |
| --- | --- | --- | --- | --- | --- | --- | --- | --- | --- |
| **Patient and reference** | **Genotype** | **Pulmonary phenotype** | **Age at pulmonary manifestations** | **First pulmonary symptoms** | **X-ray or CT diagnosis** | **Pulmonary function testing**  **Age at procedure, results (expressed as % predicted)** | **Lung biopsy** | **Treatment** | **Follow-up** |
| Nattes_2017_P9 | c.572G>T | Interstitial lung disease without neonatal RDS, chronic respiratory insufficiency | 4mo | ILD | CT: paraseptal emphysema, widespread cystic airspaces, diffuse ground glass opacities | 8 years  BGA: hypoxemia  FEV1 45  FVC 45 | y; normal findings | O_2_ supplementation (10y) | dyspnoea at rest |
| Nattes_2017_P10  Borie_2021_P1 | c.267dup | Interstitial lung disease without neonatal RDS, lung cancer | 40y | ILD , fibrosis | NA | 42 years  FEV1 44  FVC48  TLC 54  DLCO 28 | invasive mucinous adenocarcinoma | Systemic steroids, azithromycin, pirfenidone; nintedanib, received chemotherapy with carboplatin and pemetrexed | Listed for DLTX |
| Nattes_2017_P11 | c.463+2T>C | Interstitial lung disease without neonatal RDS, | 25y | ILD with resp. Insufficiency | NA | 25 years  BGA: hypoxemia  FEV1 63  FVC 72  TLC 95  DLCO 71 | No | Oral steroids (2mo), azithromycin, HCQ | hypoxemia, dyspnoea |
| Nattes_2017_P12 | c.175_176del | Interstitial lung disease without neonatal RDS, | 30y | ILD | NA | 18 years  BGA: normal  FEV1 80  FVC88  TLC 93  DLCO 66 | No | NA | Paucisymptomatic |
| Nattes_2017_P14 | Del14q13q13 | neonatal RDS progressing to ILD | neonatal | neonatal RDS | NA | 18 years  FEV1: 84,  FVC: 120  TLC: 123  DLCO: 93 | No | Invasive ventilation, oral steroids | Dyspnea at exertion |
| Nevel_2016_S1 (Young_2013_P1) | c.572G>T | Interstitial lung disease without neonatal RDS, chronic respiratory insufficiency, recurrent infections | 4mo | tachypnoea, hypoxia | CT: diffuse patchy GGO, mosaicism | 25 years  FEV1 51  FVC48  TLC 89  DLCO 83  6MWT desaturation | near normal architecture, mild peribronchial lymphocytic aggregates, increased neuroendocrine cells upon bombesin stain | O_2_ supplementation  (17y) | exercise intolerance, crackles |
| Nevel_2016_S2 | c.572G>T | Interstitial lung disease without neonatal RDS, recurrent infections | NA | tachypnoea, hypoxia | CT: subtle mosaicism lower lobes, lingula and middle lobe | 61 years  FEV1 98  FVC 99  TLC 99  DLCO 102 | No | Short-term antibiotic treatment, ICS | exercise intolerance |
| Nevel_2016_S3 | c.572G>T | Interstitial lung disease without neonatal RDS, chronic respiratory insufficiency, recurrent infections | NA | tachypnoea, hypoxia | CT: small subpleural calcified nodules with pleural thickening, subtle mosaicism | 32 years  FEV1 73  FVC 74  TLC 110  DLCO 97 | NA | O_2_ supplementation  (4y) | asymptomatic since age of 20y |
| Nevel_2016_S4 | c.572G>T | Interstitial lung disease without neonatal RDS, recurrent infections | NA | tachypnoea, hypoxia | CT: subpleural nodules and calcifications, pleural abnormalities with thickening along major fissure, nodularity of the pleura, subtle mosaicism | 61 years  FEV1 73  FVC 86  TLC 86  DLCO 91 | No | Short-term antibiotics | minimal exercise intolerance |

Abbreviations: FEV1: Forced Expiratory Volume in 1 second, FVC: Forced Vital Capacity, TLC: Total Lung Capacity, DLCO: Diffusing Capacity of the Lung for Carbon Monoxide
