# Supplementary material for: Respiratory and other organ manifestations in NKX2-1-related disorders: a systematic review
Source: Front Med (Lausanne). 2025 May 6;12:1507513. doi: 10.3389/fmed.2025.1507513 (PMC12090872; doi:10.3389/fmed.2025.1507513)
Supplement: Supplementary file 6 [file Supplementary_file_6.docx]

| **Supplementary Data 6.** Lung transplantation in *NKX2-1*-RD | | | | | | | | | |
| --- | --- | --- | --- | --- | --- | --- | --- | --- | --- |
| **Patient and reference** | **Genotype** | **Pulmonary phenotype – Age at DLTX** | **Age at pulmonary manifestations** | **First pulmonary symptoms** | **X-ray or CT diagnosis** | **Other diagnosis procedure** | **Lung biopsy** | **Treatment** | **Follow-up** |
| Hamvas_2012_PI | c.583C>T | Neonatal RDS progressing to ILD, recurrent infections, chronic respiratory insufficiency, pulmonary hypertension  8 months | neonatal | neonatal RDS (refractory) | NA | NA | growth abnormality with alveolar enlargement and simplification | O_2_ supplementation (8mo) | Death due to PH |
| Hamvas_2012_PJ | c.590T>C | Neonatal RDS progressing to ILD, recurrent infections, chronic respiratory insufficiency  10 months | neonatal | neonatal RDS | NA | NA | Representative of a surfactant dysfunction mutation with hyperplastic Type 2 pneumocytes, alveolar macrophage accumulation, and interstitial thickening | O_2_ supplementation  (10mo) | Good |
| Hamvas_2012_PK | c.592T>C | Interstitial lung disease without neonatal RDS, chronic respiratory insufficiency  13 years | 12mo | hypoxemia, ILD | NA | NA | mild to moderate alveolar growth disorder, marked chronic lobular remodelling, focal foamy macrophages, focal mild alveolar epithelial hyperplasia | NA | death due to rejection |
| Hamvas_2012_PN | c.804_812dupCGGCGGGGG | Neonatal RDS progressing to ILD, chronic respiratory insufficiency  7 months | Neonatal | neonatal RDS (refractory) | NA | NA | growth abnormality with alveolar enlargement and simplification | O_2_ supplementation | asymptomatic |
| Hamvas_2012_PS | c.1157_63dupACTACGG | Neonatal RDS progressing to ILD, recurrent infections, chronic respiratory insufficiency  22 months | Neonatal | neonatal RDS | CT: diffuse ground glass opacification and patchy consolidation | NA | severe chronic lobular remodelling with diffuse foamy macrophages, diffuse alveolar epithelial hyperplasia | O_2_ supplementation | asymptomatic |
