# Supplementary material for: Respiratory and other organ manifestations in NKX2-1-related disorders: a systematic review
Source: Front Med (Lausanne). 2025 May 6;12:1507513. doi: 10.3389/fmed.2025.1507513 (PMC12090872; doi:10.3389/fmed.2025.1507513)
Supplement: Supplementary file 7 [file Supplementary_file_7.docx]

**Supplementary Data 7.** Quality assessment results of the included studies. Table data based on Murad et al., 2018.

|  |  | **Cases series /** | | **Case reports** | | | |  |  |  |
| --- | --- | --- | --- | --- | --- | --- | --- | --- | --- | --- |
| **Domain** | **Selection** | **Ascertainment** | | **Causality** | | | | **Reporting** | **Final score** | **Quality** |
|  | **1** | **2** | **3** | **4** | **5** | **6** | **7** | **8** |  |  |
| **Asmus, 2005** | 0 | 0 | 1 | 0 | 0 | 0 | 0 | 0 | 1 | Poor |
| **Barnett, 2012** | 0 | 0 | 1 | 0 | 0 | 0 | 0 | 0 | 1 | Poor |
| **Carré, 2009** | 0 | 0 | 1 | 0 | 0 | 0 | 1 | 0 | 2 | Poor |
| **Devos, 2006** | 0 | 0 | 1 | 0 | 0 | 0 | 0 | 0 | 1 | Poor |
| **Doyle, 2004** | 0 | 0 | 1 | 0 | 0 | 0 | 1 | 0 | 2 | Poor |
| **Ferrara, 2008** | 0 | 0 | 0 | 0 | 0 | 0 | 0 | 0 | 0 | Poor |
| **Ferrara, 2012** | 1 | 0 | 1 | 0 | 0 | 1 | 0 | 0 | 3 | Medium |
| **Galambos, 2010** | 0 | 1 | 1 | 0 | 0 | 1 | 1 | 1 | 5 | Good |
| **Gillett, 2013** | 0 | 0 | 1 | 0 | 0 | 0 | 0 | 0 | 1 | Poor |
| **Glik, 2008** | 0 | 0 | 0 | 0 | 0 | 0 | 0 | 0 | 0 | Poor |
| **Gras, 2012** | 1 | 0 | 1 | 0 | 0 | 0 | 0 | 0 | 2 | Poor |
| **Gu, 2020** | 0 | 0 | 1 | 0 | 0 | 0 | 0 | 0 | 1 | Poor |
| **Guillot, 2010** | 0 | 1 | 1 | 0 | 0 | 0 | 1 | 1 | 4 | Medium |
| **Hamvas, 2013** | 1 | 0 | 1 | 0 | 0 | 0 | 1 | 0 | 3 | Medium |
| **Hanes, 2018** | 0 | 0 | 1 | 0 | 0 | 0 | 0 | 0 | 1 | Poor |
| **Hu, 2019** | 0 | 0 | 1 | 0 | 0 | 0 | 0 | 0 | 1 | Poor |
| **Iwatani, 2000** | 0 | 0 | 1 | 0 | 0 | 0 | 1 | 0 | 2 | Poor |
| **Jovien, 2016** | 0 | 1 | 1 | 0 | 1 | 0 | 1 | 0 | 4 | Medium |
| **Kleinlein, 2010** | 0 | 0 | 1 | 1 | 0 | 0 | 1 | 0 | 3 | Medium |
| **Koht, 2016** | 0 | 0 | 1 | 0 | 0 | 0 | 0 | 0 | 1 | Poor |
| **LeMoine, 2019** | 1 | 0 | 1 | 0 | 0 | 0 | 0 | 0 | 2 | Poor |
| **Lynn, 2020** | 0 | 0 | 1 | 0 | 0 | 0 | 1 | 0 | 2 | Poor |
| **Maquet, 2009** | 0 | 0 | 0 | 1 | 0 | 0 | 0 | 0 | 1 | Poor |
| **Maric, 2020** | 0 | 0 | 1 | 0 | 0 | 0 | 1 | 0 | 2 | Poor |
| **Mirza, 2022** | 0 | 1 | 1 | 1 | 0 | 0 | 1 | 0 | 4 | Medium |
| **Nattes, 2017** | 1 | 0 | 1 | 0 | 0 | 0 | 1 | 0 | 3 | Medium |
| **Nevel, 2016** | 1 | 0 | 1 | 0 | 0 | 0 | 0 | 0 | 2 | Poor |
| **Parnes, 2019** | 1 | 0 | 1 | 0 | 0 | 0 | 0 | 0 | 2 | Poor |
| **Peall, 2014** | 0 | 0 | 1 | 0 | 0 | 0 | 0 | 0 | 1 | Poor |
| **Pohlenz, 2002** | 0 | 0 | 0 | 0 | 0 | 0 | 0 | 0 | 0 | Poor |
| **Prasad, 2019** | 0 | 0 | 1 | 0 | 0 | 0 | 1 | 0 | 2 | Poor |
| **Safi, 2017** | 0 | 0 | 1 | 0 | 0 | 0 | 0 | 0 | 1 | Poor |
| **Salerno, 2014** | 0 | 0 | 1 | 0 | 0 | 0 | 0 | 0 | 1 | Poor |
| **Salvado, 2013** | 0 | 0 | 1 | 0 | 0 | 0 | 1 | 0 | 2 | Poor |
| **Sutton, 2022** | 1 | 0 | 0 | 0 | 0 | 0 | 0 | 0 | 1 | Poor |
| **Thorwarth, 2014** | 1 | 0 | 1 | 0 | 0 | 0 | 0 | 0 | 2 | Poor |
| **Villamil-Osorio, 2021** | 0 | 0 | 1 | 0 | 0 | 0 | 1 | 0 | 2 | Poor |
| **Willemsen, 2005** | 0 | 0 | 1 | 0 | 0 | 0 | 0 | 0 | 1 | Poor |
