# Supplementary material for: Respiratory and other organ manifestations in NKX2-1-related disorders: a systematic review
Source: Front Med (Lausanne). 2025 May 6;12:1507513. doi: 10.3389/fmed.2025.1507513 (PMC12090872; doi:10.3389/fmed.2025.1507513)
Supplement: Supplementary file 8 [file Supplementary_file_8.docx]

Medline search for the Systematic review about diagnosis

What are the best procedures for the diagnosis of lung diseases in patients with NKX2-1-related disorders?

Database: Ovid MEDLINE(R) and Epub Ahead of Print, In-Process, In-Data-Review & Other Non-Indexed Citations and Daily <1946 to August 11, 2022>

Search Date: 12/08/2022

Search Strategy:

--------------------------------------------------------------------------------

1 Chorea/ or (chorea* or "Brain-Lung-Thyroid Syndrome" or "brain-lung-thyroid" or "brain-thyroid-lung").ti,ab. (8289)

2 thyroid nuclear factor 1/ or (thyroid nuclear factor 1 or thyroid transcription factor 1).ti,ab. (2335)

3 ('titf1' or 'titf 1' or 'titf-1' or 'ttf1' or 'ttf 1' or 'ttf-1').ti,ab. (2589)

4 ('nkx2 1' or 'nkx2-1' or 'nkx2?1' or 'nkx 2 1' or 'nkx 2-1' or 'nkx 2?1' or 'nk2 homeobox 1 protein' or 'nkx2 homeodomain transcription').ti,ab. (885)

5 PAX9 Transcription Factor/ or Chromosomes, Human, Pair 14/ge [Genetics] (1887)

6 ('pax9 transcription factor' or 'paired box 9 protein' or 'paired box transcription factor 9' or 'pax9' or 'pax-9' or 'pax 9' or 'pair box 9' or 'chromosome 14' or 14q12* or 14q13*).ti,ab. (3072)

7 1 or 2 or 3 or 4 or 5 or 6 (16597)

8 exp Lung diseases/ or ((pulmonar* or lung$) adj3 disease$).ti,ab. (1193256)

9 exp Respiratory Distress Syndrome, Newborn/ or exp Bronchopulmonary Dysplasia/ or exp Hyaline Membrane Disease/ (20547)

10 (((infantile or neonatal or infant or newborn) adj3 'respiratory distress syndrome') or NRDS or "Neonatal hyaline membrane disease" or NHMD).ti,ab. (2061)

11 ('Chronic Lung Disease' or 'Respiratory Distress Syndrome' or RDS or BPD).ti,ab. (53954)

12 8 or 9 or 10 or 11 (1214636)

13 exp Acute Lung Injury/ or exp Respiratory Distress Syndrome/ (45684)

14 ((acute adj4 (lung injur* or distress syndrome)) or ALI).ti,ab. (36200)

15 ((severe or hypoxic) adj4 'respiratory failure').ti,ab. (4328)

16 13 or 14 or 15 (67430)

17 exp Lung Diseases, Interstitial/ (82228)

18 ((interstitial$ adj3 (lung$ or pulmonar* or pneumon* or pneumopath*)) or ILD).ti,ab. (28320)

19 (((pulmonar* or lung$) adj3 (fibros* or fibrot*)) or sarcoidos?s or alveolit*).ti,ab. (58783)

20 17 or 18 or 19 (129072)

21 "Transient Tachypnea of the Newborn"/ (151)

22 (newborn adj3 (transient or transitory) adj3 tachypnea$).ti,ab. (63)

23 Neuroendocrine Cells/ and Hyperplasia/ (131)

24 (((Cell* adj3 Neuroendocrine) or (Endocrine adj3 Neuron*)) and hyperplasia*).ti,ab. (484)

25 23 or 24 (502)

26 Pneumonia/ or Respiratory Tract Infections/ (92245)

27 ((respirator* or pulmonar*) adj3 (infect* or inflam* or diseas* or illness*)).ti,ab. (218330)

28 (pneumon* or bronchopneumon* or pleuropneumon*).ti,ab. (224784)

29 (lower respiratory tract infection* or lower respiratory infection* or lrti).ti,ab. (9122)

30 ("gram negative bacilli" or "pseudomonas a?ruginosa" or enterobacter* or "lung inflammation*").ti,ab. (110440)

31 26 or 27 or 28 or 29 or 30 (533190)

32 exp Pulmonary Surfactants/ (13881)

33 (Surfactant* adj3 (Pulmonar* or alveol*)).ti,ab. (5191)

34 (Surfactant* adj3 (deficienc* or disorder* or disruption*)).ti,ab. (925)

35 32 or 33 or 34 (15492)

36 Respiratory Insufficiency/ (34979)

37 (respiratory fail* adj3 (lethal or mortal)).ti,ab. (69)

38 exp Hypoxia/ (86757)

39 ((Oxygen adj3 deficiency) or hypoxia or hypoxem* or hypercapnia or AHRF).ti,ab. (153070)

40 exp Lung Neoplasms/ (263625)

41 ((lung* or pulmonary or bronch*) adj3 (cancer* or neoplasm* or carcinoma* or tumo#r* or lymphoma* or metast* or malignan* or blastoma* or carcinogen* or adenocarcinoma* or angiosarcoma* or chrondosarcoma* or sarcoma* or teratoma* or microcytic*)).ti,ab. (291856)

42 ((pancoast* or superior sulcus or pulmonary sulcus) adj4 (tumo#r* or syndrome*)).tw. (395)

43 ((lung* or pulmonary or bronch*) adj4 (oat or small or non-small) adj4 cell*).tw. (91992)

44 (SCLC or NSCLC).tw. (61244)

45 Carcinoma, Non-Small-Cell Lung/ (65771)

46 (((Bronch* or Lung$ or Pulmon*) adj ("Non Small Cell$" or "Nonsmall Cell$") adj (Cancer* or Carcino*)) or (("Non Small Cell$" or "Nonsmall Cell$") adj (Bronch* or Lung$ or Pulmon*) adj (Cancer* or Carcino*))).ti,ab. (78148)

47 Carcinoma, Large Cell/ or large cell carcinoma.ti,ab. (3912)

48 Lung/ or lung$.ti,ab. (830151)

49 47 and 48 (3401)

50 Pulmonary Alveolar Proteinosis/ (1763)

51 alveolar proteinos?s pulmonary.ti,ab. (5)

52 Bronchitis, Chronic/ (1864)

53 (chronic* adj3 bronchiti*).ti,ab. (11479)

54 (obstruct* adj3 (pulmonar* or lung$ or airwa* or airflow$ or bronch* or respirat*)).ti,ab. (98065)

55 exp Asthma/ (138657)

56 (Asthma or Wheez*).ti,ab. (166617)

57 Bronchial Spasm/ (4346)

58 ((bronchial adj1 spasm*) or bronchospas*).ti,ab. (5805)

59 Bronchoconstriction/ (4273)

60 (Bronchoconstriction$ or (Bronch* adj3 Constriction*)).ti,ab. (8181)

61 Bronchial Hyperreactivity/ (7521)

62 (Bronchial adj3 Hyperreactivit*).ti,ab. (2208)

63 Reactive airway disease.ti,ab. (351)

64 Bronchopulmonary Dysplasia/pp [Physiopathology] (947)

65 Lung/gd, pp [Growth & Development, Physiopathology] (27986)

66 *Organogenesis/ or Histology/ or (histopathology or histology or organogenesis).ti,ab. (214330)

67 ((lung$ adj1 (lesion$ or malformation$)) or 'disrupted lung growth').ti,ab. (7006)

68 64 or 65 or 66 or 67 (249178)

69 12 or 16 or 20 or 21 or 22 or 25 or 31 or 35 or 36 or 37 or 38 or 39 or 40 or 41 or 42 or 43 or 44 or 45 or 46 or 49 or 50 or 51 or 52 or 53 or 54 or 55 or 56 or 57 or 58 or 59 or 60 or 61 or 62 or 63 or 68 (2082242)

70 7 and 69 [Population] (2572)

71 exp Physical Examination/ (1331735)

72 ((physical or medical or clinical) adj3 (exam* or check* or assess*)).ti,ab,kf. (340567)

73 exp Blood Chemical Analysis/ or exp Hematologic Tests/ or Blood Culture/ (398362)

74 (Oximet* or "peak flow" or "pulse ox" or hemogram$ or (blood adj4 (monitor* or test* or analys* or count* or culture$ or sampl*))).ti,ab,kf. (434751)

75 C-Reactive Protein/ (51868)

76 ("C reactive protein" or hsCRP).ti,ab,kf. (85169)

77 exp Diagnostic Techniques, Respiratory System/ or Exercise Tolerance/ or Carbon Monoxide/ (327771)

78 (((Pulmon* or lung$ or bronch* or respirator* or exercise* or "carbon monoxide") adj4 (monitor* or test* or analys* or count* or measur* or culture$)) or DLCO).ti,ab,kf. (176879)

79 exp Bronchoalveolar Lavage/ (29989)

80 ((bronch* or lung$ or pulmon*) adj4 lavage$).ti,ab,kf. (39800)

81 exp Diagnostic Imaging/ (2858749)

82 (X-ray$ or Xray$ or scan$ or radiograph* or tomograph* or Ultraso* or PET$ or CT$ or MRI$ or "magnetic resonance" or angiogram$).ti,ab,kf. (2707201)

83 exp Cytological Techniques/ (1567274)

84 (Cytolog* or cytomet* or biops*).ti,ab,kf. (725771)

85 exp Diagnostic Techniques, Respiratory System/ or exp Endoscopy/ (654992)

86 (Bronchograph* or Bronchoscop* or Capnograph* or Plethysmograph* or Spirometr* or Bronchospirometr* or ((pulmonar* or test$) adj3 (ventilation or expirator*))).ti,ab,kf. (79480)

87 or/71-86 [Diagnosis] (8182628)

88 and/70,87 (1530)

89 animals/ not humans/ (5002133)

90 (letter or "historical article*" or (comment or editorial or in vitro or news)).pt,ti. (3020819)

91 89 or 90 (7815429)

92 88 not 91 (1403)

93 limit 92 to yr="2002 -Current" (1308)

Medline Search for the Systematic review about treatment and follow-up

What are the best procedures for treatment and follow-up of lung diseases in patients with NKX2-1 related disorders?

Search Date: 12/08/2022

Database: Ovid MEDLINE(R) and Epub Ahead of Print, In-Process, In-Data-Review & Other Non-Indexed Citations and Daily <1946 to August 12, 2022>

Search Strategy:

--------------------------------------------------------------------------------

1 Chorea/ or (chorea* or "Brain-Lung-Thyroid Syndrome" or "brain-lung-thyroid" or "brain-thyroid-lung").ti,ab. (8289)

2 thyroid nuclear factor 1/ or (thyroid nuclear factor 1 or thyroid transcription factor 1).ti,ab. (2335)

3 ('titf1' or 'titf 1' or 'titf-1' or 'ttf1' or 'ttf 1' or 'ttf-1').ti,ab. (2589)

4 ('nkx2 1' or 'nkx2-1' or 'nkx2?1' or 'nkx 2 1' or 'nkx 2-1' or 'nkx 2?1' or 'nk2 homeobox 1 protein' or 'nkx2 homeodomain transcription').ti,ab. (887)

5 PAX9 Transcription Factor/ or Chromosomes, Human, Pair 14/ge [Genetics] (1887)

6 ('pax9 transcription factor' or 'paired box 9 protein' or 'paired box transcription factor 9' or 'pax9' or 'pax-9' or 'pax 9' or 'pair box 9' or 'chromosome 14' or 14q12* or 14q13*).ti,ab. (3073)

7 1 or 2 or 3 or 4 or 5 or 6 (16600)

8 exp Lung diseases/ or ((pulmonary or lung) adj3 disease?).ti,ab. (1193099)

9 exp Respiratory Distress Syndrome, Newborn/ or exp Bronchopulmonary Dysplasia/ or exp Hyaline Membrane Disease/ (20554)

10 (((infantile or neonatal or infant or newborn) adj3 'respiratory distress syndrome') or NRDS or "Neonatal hyaline membrane disease" or NHMD).ti,ab. (2066)

11 ('Chronic Lung Disease' or 'Respiratory Distress Syndrome' or RDS or BPD).ti,ab. (53983)

12 exp Acute Lung Injury/ or exp Respiratory Distress Syndrome/ (45696)

13 ((acute adj4 (lung injur* or distress syndrome)) or ALI).ti,ab. (36219)

14 ((severe or hypoxic) adj4 (respiratory and failure)).ti,ab. (8889)

15 exp Lung Diseases, Interstitial/ (82231)

16 ((interstitial$ adj3 (lung$ or pulmonary$ or pneumon$ or pneumopathy)) or ILD).ti,ab. (28301)

17 (((pulmonary$ or lung$) adj3 (fibros$ or fibrot$)) or sarcoidosis$ or alveolitis$).ti,ab. (58780)

18 "Transient Tachypnea of the Newborn"/ (153)

19 (newborn adj3 (transient or transitory) adj3 tachypnea?).ti,ab. (63)

20 Neuroendocrine Cells/ and Hyperplasia/ (131)

21 (((Cell? adj3 Neuroendocrine) or (Endocrine adj3 Neuron?)) and hyperplasia?).ti,ab. (483)

22 Pneumonia/ or Respiratory Tract Infections/ (92258)

23 ((respiratory or pulmonary$) adj3 (infect$ or inflam$ or diseas$ or illness$)).ti,ab. (218354)

24 (pneumon* or bronchopneumon* or pleuropneumon*).ti,ab. (224876)

25 (lower respiratory tract infection* or lower respiratory infection* or lrti).ti,ab. (9126)

26 ("gram negative bacilli" or "pseudomonas a?ruginosa" or enterobacter$ or "lung inflammation").ti,ab. (110464)

27 exp Pulmonary Surfactants/ (13883)

28 (Surfactant? adj3 Pulmonary).ti,ab. (4313)

29 (Surfactant adj3 (deficiency or disorder or disruption)).ti,ab. (799)

30 exp Respiratory Insufficiency/ (67200)

31 (respiratory fail* adj3 (lethal or mortal)).ti,ab. (69)

32 exp Hypoxia/ (86769)

33 ((Oxygen adj3 Deficiency) or hypoxia or hypoxem* or hypercapnia or ahrf).tw. (153126)

34 exp Lung Neoplasms/ (263670)

35 ((lung* or pulmonary or bronch*) adj3 (cancer* or neoplasm* or carcinoma* or tumo?r* or lymphoma* or metast* or malignan* or blastoma* or carcinogen* or adenocarcinoma* or angiosarcoma* or chrondosarcoma* or sarcoma* or teratoma* or microcytic*)).ti,ab. (303191)

36 ((pancoast* or superior sulcus or pulmonary sulcus) adj4 (tumo?r* or syndrome*)).tw. (883)

37 ((lung* or pulmonary or bronch*) adj4 (oat or small or non-small) adj4 cell*).tw. (92054)

38 (SCLC or NSCLC).tw. (61296)

39 Carcinoma, Non-Small-Cell Lung/ (65786)

40 (((Bronch* or Lung or Pulmon*) adj ("Non Small Cell?" or "Nonsmall Cell?") adj (Cancer* or Carcino*)) or (("Non Small Cell?" or "Nonsmall Cell?") adj (Bronchial or Lung or Pulmon*) adj (Cancer* or Carcino*)) or NSCLC*).ti,ab. (82290)

41 Carcinoma, Large Cell/ or large cell carcinoma.ti,ab. (3912)

42 Lung/ or lung?.ti,ab. (828074)

43 41 and 42 (3400)

44 Pulmonary Alveolar Proteinosis/ (1763)

45 alveolar proteinos#s pulmonary.ti,ab. (5)

46 Bronchitis, Chronic/ (1866)

47 (chronic$ adj3 bronchiti$).ti,ab. (11479)

48 (obstruct$ adj3 (pulmonary or lung$ or airway$ or airflow$ or bronch$ or respirat$)).ti,ab. (98077)

49 exp Asthma/ (138665)

50 (Asthma or Wheez$).ti,ab. (166660)

51 Bronchial Spasm/ (4346)

52 ((bronchial adj1 spasm?) or bronchospas$).ti,ab. (5799)

53 Bronchoconstriction/ (4273)

54 ((Bronchoconstriction or Bronchial) adj3 Constriction?).ti,ab. (246)

55 Bronchial Hyperreactivity/ (7521)

56 (Bronchial adj3 Hyperreactivit*).ti,ab. (2208)

57 Reactive airway disease.ti,ab. (351)

58 Bronchopulmonary Dysplasia/pp [Physiopathology] (947)

59 Lung/gd, pp [Growth & Development, Physiopathology] (27986)

60 *Organogenesis/ or Histology/ or (histopathology or histology or organogenesis).ti,ab. (214410)

61 ((lung adj1 (lesion or malformation)) or 'disrupted lung growth').ti,ab. (1716)

62 8 or 9 or 10 or 11 or 12 or 13 or 14 or 15 or 16 or 17 or 18 or 19 or 20 or 21 or 22 or 23 or 24 or 25 or 26 or 27 or 28 or 29 or 30 or 31 or 32 or 33 or 34 or 35 or 36 or 37 or 38 or 39 or 40 or 43 or 44 or 45 or 46 or 47 or 48 or 49 or 50 or 51 or 52 or 53 or 54 or 55 or 56 or 57 or 58 or 59 or 60 or 61 (2102212)

63 7 and 62 (2594)

64 Oxygen Inhalation Therapy/ or Oxygen/ or oxygen consumption/ or ((oxygen* or o2) adj4 (supplement* or long* or prolong* or indefinit* or contin* or ongoing or timespan or duration or length*)).tw. or ((inhalation* or respiratory) adj4 therap*).tw. or LTOT.tw. (298369)

65 Respiration, Artificial/ or exp Ventilators, Mechanical/ or Positive-Pressure Respiration/ or tracheotomy/ or tracheostomy/ or exp Intubation, Intratracheal/ (123412)

66 ((artificial adj1 respiration) or (mechanical adj1 ventilator$) or respiratory insufficiency or positive pressure$ or positivepressure$ or bipap or tracheotomy or tracheostomy or intubation).tw. (90697)

67 ((assist* or support* or mandatory or controlled or invasive or non invasive or noninvasive) adj3 ventilat*).tw. (32614)

68 Drug Therapy.fs. (2519979)

69 exp Bronchodilator Agents/ (274169)

70 (bronchodilator* or bronchial dilat* or broncholytic*).ti,ab. (15146)

71 exp Adrenergic Agents/ (390829)

72 adrenergic agent$.ti,ab. (1510)

73 exp Adrenal Cortex Hormones/ (417520)

74 ((adrenal adj3 hormone?) or corticoid? or corticosteroid? or steroid? or glucocorticoid*).ti,ab. (377792)

75 exp Immunosuppressive Agents/ (337448)

76 ((Agent? adj3 Immunosuppressive) or Immunosuppressant?).ti,ab. (29424)

77 Antibiotic prophylaxis/ or exp Anti-Bacterial Agents/ (799773)

78 (antibiotic$ or anti-biotic$ or antimicrobial$ or anti-microbial$ or ((antibacterial or anti bacterial) adj3 agent*)).tw. (539520)

79 (antibiotic* adj5 (prophylaxis or prophylactic* or premedication*)).ti,ab. (18732)

80 Surface-Active Agents/ (32097)

81 (Surfactant? or (Amphiphilic adj3 Agent?) or Tenside? or (Surface adj3 Agent?)).ti,ab. (68398)

82 Respiratory Therapy/ (6960)

83 exp Physical Therapy Modalities/ (172750)

84 ((chest or thora$) adj3 (physiotherap$ or physical therap$)).ti,ab. (1174)

85 Drainage, Postural/ (254)

86 (postur$ adj3 drain$).ti,ab. (414)

87 Percussion/ (1087)

88 ((chest or thora$) adj3 percuss$).ti,ab. (168)

89 Vibration/ (27093)

90 Chest Wall Oscillation/ (334)

91 vibrat$.ti,ab. (87532)

92 ((chest or thora$) adj3 (shak$ or oscillat$)).ti,ab. (180)

93 Breathing Exercises/ (3788)

94 ((direct$ or induc$ or provok$ or assist$) adj3 cough$).ti,ab. (2174)

95 ((forced or passive or compress$ or prolong$ or slow$ or accelerat$ or increas$) adj3 (exhal$ or expirat$)).ti,ab. (27354)

96 (breath$ adj3 exercis$).ti,ab. (3002)

97 Lung Transplantation/ (17142)

98 ((lung* or pulmonary) adj4 (transplant* or grafting* or allotransplant*)).tw. (23347)

99 ((endobronchial or intrabronchial or intra bronchial) adj4 (nitinol or coil* or valve* or spring* or spiral*)).tw. (538)

100 (LVR or LVRS or LVRC).tw. (1132)

101 exp Surgical Oncology/ (694)

102 "Surgical Oncology".tw. (2689)

103 ((surg* or operat* or microsurg* or resect* or dissect* or microdissect* or excis or microresect*) adj3 (neoplasm$ or cancer$ or carcinoma$ or adenocarcinoma$ or angiosarcoma$ or chrondosarcoma$ or sarcoma$ or teratoma$ or lymphoma$ or blastoma$ or microcytic$ or carcinogenesis or tumour$ or tumor$ or metast$)).ti,ab. (194913)

104 Drug Therapy/ or exp Drug Therapy, Combination/ or exp Antineoplastic Protocols/ or exp Antineoplastic Agents/ or Chemotherapy, Adjuvant/ (1455137)

105 ('drug therap*' or chemotherap* or antineoplastic* or anti-neoplastic* or polychemotherap* or CTX).tw. (535324)

106 ((anticancer* or anti-cancer* or antitumo?r or anti-tumo?r or anticarcinogen* or anti-carcinogen*) adj4 (drug* or agent* or therap* or treat* or medicat* or protocol*)).tw. (126370)

107 exp Nutrition Therapy/ or Food/ or Diet/ or Dietetics/ or (food or beverage* or drink* or water or nutrition* or nutrient* or diet*).ti,ab. (2300804)

108 exp Immunization/ or Immunotherapy, Active/ or Cancer Vaccines/ or Vaccines/ or (vaccin* or immunotherap*).ti,ab. (561144)

109 ((immunothe* or immuniz* or vaccin*) and (activ* or therap* or treat*)).ti,ab. (235163)

110 Environment/ or (Environment* or ((Impact? or care) adj1 Environmental)).ti,ab. (1240504)

111 (((best or optim$) adj2 support$ adj2 (care or treatment$)) or supportive care).ti,ab. (18802)

112 64 or 65 or 66 or 67 or 68 or 69 or 70 or 71 or 72 or 73 or 74 or 75 or 76 or 77 or 78 or 79 or 80 or 81 or 82 or 83 or 84 or 85 or 86 or 87 or 88 or 89 or 90 or 91 or 92 or 93 or 94 or 95 or 96 or 97 or 98 or 99 or 100 or 101 or 102 or 103 or 104 or 105 or 106 or 107 or 108 or 109 or 110 or 111 (9143636)

113 63 and 112 (867)

114 (letter or "historical article*" or (comment or editorial or in vitro or news)).pt. (2624556)

115 113 not 114 (856)

116 animals/ not (animals/ and humans/) (5002581)

117 115 not 116 (768)

118 limit 117 to yr="2002 -Current" (672)

***************************
